# Post-stroke pneumonia at the stroke unit – a registry based analysis of contributing and protective factors

**DOI:** 10.1186/s12883-016-0627-y

**Published:** 2016-07-18

**Authors:** Karl Matz, Leonhard Seyfang, Alexandra Dachenhausen, Yvonne Teuschl, Jaakko Tuomilehto, Michael Brainin, MD for the Austrian Stroke Unit Registry Collaborators

**Affiliations:** Department for Clinical Neurosciences and Preventive Medicine, Danube University Krems, Dr.-Karl-Dorrek-Str. 30, A-3500 Krems, Austria; Department of Neurology, University Clinic Tulln, Karl Landsteiner Privatuniversität für Gesundheitswissenschaften, Krems, Austria; Department of Chronic Disease Prevention, National Institute for Health and Welfare, 00271 Helsinki, Finland; Diabetes Research Group, King Abdulaziz University, 21589 Jeddah, Saudi Arabia

**Keywords:** Acute ischemic stroke, Stroke unit, Pneumonia, Respiratory infection, Risk factors

## Abstract

**Background:**

To investigate prevalence and risk factors for post stroke pneumonia (PSP) in patients with acute ischemic stroke treated at stroke units (SU).

**Method:**

We analysed data from the Austrian Stroke Unit registry concerning admissions from January 2003 to December 2013 and assessed the prevalence of PSP at the stroke unit. Patients with and without PSP were compared in univariate and multivariate models searching for factors associated with the occurrence of PSP at the SU.

**Results:**

Three thousand one hundred eleven patients (5.2 %) of 59,558 analysed patients were diagnosed with PSP. While age and stroke severity were non-modifiable factors associated with PSP, modifiable risk factors included chronic alcohol consumption and atrial fibrillation. Patients who developed neurological, cardiac, and other infective complications showed a higher prevalence of PSP, an increased prevalence was also found in connection with the placement of nasogastric tubes or urinary catheters. Female sex, left hemispheric stroke, cryptogenic stroke pathogenesis and additionally, treatment with lipid lowering drugs were factors associated with a lower PSP prevalence.

**Conclusion:**

Pneumonia in acute ischemic stroke is associated with a variety of modifiable and unmodifiable factors that allow to identify patients at high risk of developing PSP and to focus on early preventive measures at the SU. Further studies could use the results of this study to explore potential benefits of specific interventions targeted at these factors.

**Electronic supplementary material:**

The online version of this article (doi:10.1186/s12883-016-0627-y) contains supplementary material, which is available to authorized users.

## Background

Pneumonia is a frequently encountered problem affecting stroke patients. Whereas in stroke registries, a prevalence of post stroke pneumonia (PSP) from 2 % [1] to 7.5 % [2] has been found, among patients participating in randomized controlled trials PSP was encountered in 10 to 14 % [3, 4]. In prospective cohorts of patients with acute stroke chest infection occurred with a prevalence between 7.4 and 12 % [5]. A meta-analysis [6] covering 87 studies with 137817 patients found an overall prevalence of 10 %. In a large Chinese cohort [7] pneumonia was the most common medical complication with a prevalence of 11.4 %. A significantly higher prevalence of PSP has been found in cohorts of thrombolysed stroke patients [8] and patients treated at intensive care units (ICUs) [9, 10], reaching the highest rate with 28 % among artificially ventilated patients [11].

In a metaanalysis [12] of studies from 2000 to 2015 the five most important risk factors for development of PSP were: multiple vertebrobasilar stroke, National Institutes of Health Stroke Scale score (NIHSS) >15 points, mechanical ventilation, nasogastric tube use and dysphagia. Other risk factors found in case control studies were age [13, 14], dysarthria [12], chronic lung disease [13] or pretreatment with proton pump inhibitors [15]. In respect of anatomical characteristics lesion size [16], basal ganglia infarction [17] and hemorrhagic transformation of infarction were factors predisposing to PSP.

Aspiration due to dysphagia is thought to be a key component of the pathogenesis of PSP. A metaanalysis of seven studies with 891 patients has shown that dysphagia confers a more than threefold increased relative risk (RR) for PSP, dysphagia with aspiration in swallowing test a more than tenfold increased risk [18].

PSP is associated with reduced functional outcome [19], prolonged stay in hospital [20] and increased risk of death [21]. The pooled odds ratio for in-hospital mortality was 3.62 in a metaanalysis [6] of four studies and a recent study found PSP to be the medical complication most strongly associated with death or dependency after recurrent stroke [22].

### Purpose of the study

The objective of the study was to analyse and compare patients with and without pneumonia during the stay at the SU in order to find relevant patients characteristics or stroke unit procedures predisposing to or being associated with PSP.

## Methods

The study was designed as a case–control type analysis of data from the Austrian Stroke Unit Registry (ASUR) that prospectively collects data from patients with acute stroke admitted to SUs in Austria nationwide. The setup and method of this registry have been previously described [23].

In short, the ASUR is a prospective online registry of all acute stroke patients admitted to the SU network. It comprises records of stroke patients from meanwhile 38 Austrian SUs that have been collected continuously since 2003. The ASUR is financed by the Federal Ministry of Health and is centrally administered by the Gesundheit Österreich GmbH. Participating Austrian SUs document anonymized stroke-relevant data in the registry. All Scientific analyses are approved and supervised by a steering committee. Quality is regularly assessed by online analysis and national SU user and benchmarking meetings. For each SU admission data on stroke onset, severity, and subtype, known risk factors, imaging modalities, complications, treatments and outcome variables are recorded. Vascular risk factors were determined by reviewing medical history and prestroke medication, or assessed and newly diagnosed during the stay at the SU. The risk factors recorded were hypertension, hypercholesterolemia, diabetes mellitus, previous stroke, atrial fibrillation, myocardial infarction, other cardiac diseases, peripheral arterial disease, smoking and regular alcohol consumption. Hypertension was defined by a history of known hypertension with a systolic blood pressure ≥140 mmHg or diastolic blood pressure ≥90 mmHg (according to the criteria of the Seventh Report of Joint National Committee on Prevention and Detection, Evaluation, and Treatment of High Blood Pressure) [24], or a presence of antihypertensive drug treatment. Hypercholesterolemia was defined by a history of total cholesterol level ≥200 mg/dL or in the blood sample taken during hospital stay or a presence of lipid-lowering therapy. Lipid lowering treatment status was entered positive if either treatment was continued or newly prescribed. Diabetes mellitus was defined according to the World Health Organization [25] if the fasting plasma glucose level ≥126 mg/dL or 2 h plasma glucose level ≥200 mg/dL have been reported in the medical history or if patients were treated with insulin or oral hypoglycaemic agents. Smoking was defined as any habitual, not casual, consumption of cigarettes, pipes or cigars. Regular alcohol consumption (moderate or more) was defined as estimated average consumption >1 standard drink per day (>500 mL beer or >250 mL wine) according to national guidelines [26]. Atrial fibrillation, if not known before stroke, was diagnosed using ECG monitoring on the SU. Stroke types were classified based on neuroimaging (CT or MRI) findings according to the International Classification of Diseases (ICD)-10 code into ischemic stroke (I63) or primary intracerebral haemorrhage (I61). Stroke cause was classified into five subtypes (macroangiopathy, cardioembolism, microangiopathy, other determined etiology, and undetermined etiology) according to the Trial of Org 10172 in Acute Stroke Treatment (TOAST) criteria [27]. National Institute of Health Stroke Scale (NIHSS) was used to determine clinical severity of the stroke event on admission and at discharge from the SU. Functional status was measured by Barthel Index and modified Rankin scale on admission, at discharge from the SU and at follow-up after three months. The follow-up was done by telephone interview of patients or caregivers in most cases.

Pneumonia was recorded as one of several clinical complications during the stay at the SU and was classified as such by the treating physician usually based on clinical symptoms of lung infection in combination with clinical sings such as rales on chest auscultation and chest X ray findings suggestive for pneumonia supported by elevation of inflammatory markers in laboratory tests. This follows in approximation the recently proposed adapted Centers for Disease Control and Prevention guidelines for the diagnosis of stroke associated pneumonia [28].

### Statistical methods

Group differences were tested with the Chi-square test for independence in case of categorical variables and with the Wilcoxon rank sum test for equality of the location parameter for numeric variables. A multivariate logistic regression model assessed the relationship between the dichotomous target variable PSP and various explanatory variables. Variable selection was done by the bidirectional stepwise variable selection procedure, optimizing the Bayesian information criterion (BIC). The start model was the trivial model, including just the intercept. All potential explanatory variables that were available for the variable selection are listed in Additional file 1: Table S1. The final model, resulting from the variable selection procedure, is also given in Additional file 1: Table S1. All data were processed using the statistical environment R, version 2.15.2 [29].

The second model was restricted to all cases whose status of lipid lowering therapy has been entered into the data registry since 2007 (*n* = 47321), thus 12237 patients were excluded. Explanatory variables of this model are listed in Additional file 1: Table S2. A description of the data selection process with frequencies of included variables, univariate comparisons and multivariate regression models is provided with Additional file 2.

## Results

From 2003 until December 2013, data from 68583 patients with acute ischemic stroke were entered into the ASUR. Complete data sets for the SU period were available for 59558 (87 %) patients of whom 3111 (5.2 %) had PSP during the stay at the SU. The mean (or median) length of stay at the SU was 4.2 days for all patients. Clinical and demographic variables are listed in Table 1.

**Table 1:** Risk factors

	Pneumonia at the SU					
	No	Yes	Univariate analysis		Multivariate analysis	
Patient characteristics	56447 (94.8 %)	3111 (5.2 %)	P	5–95 % CI	OR	*p*
Age, y, median (interquartile range)	72.9 (62.8, 81.3)	79.8 (72, 85.1)	<0.001		Age 55–64	
				1.54–2.39	1.92	<0.001
					Age 75–84	
				1.38–1.67	1.52	<0.001
Female sex, n (%)	26521 (47)	1457 (46.8)	0.87	0.51–0.61	0.56	<0.001
Time onset-admission, median (interquartile range)	120 (61, 280)	90 (57, 156)	<0.001			
Stroke syndrome, n (%)						
LACS	17592 (31.2)	362 (11.6)	<0.001			
PACS	21627 (38.3)	1214 (39	0.428			
TACS	5678 (10.1)	1069 (34.4)	<0.001			
POCS	9555 (16.9)	386 (12.4)	<0.001			
mRS 0 before stroke, n (%)	39601 (70.2)	1507 (48.4)	<0.001			
NIHSS on admission, (median, interquartile range)	3 (1, 7)	14 (8, 20)	<0.001			
Left hemisphere	25692 (45.5)	1446 (46.5)	0.293	0.78–0.93	0.85	<0.001
Right hemisphere	19853 (35.2)	1250 (40.2)	<0.001			
Both hemispheres	987 (1.7)	67 (2.2)	0.095			
Brainstem or cerebellum	9915 (17.6)	348 (11.1)	<0.001			
Atrial Fibrillation, n (%)	14031 (24.9)	1596 (51.3)	<0.001	1.15–1.38	1.26	<0.001
Previous stroke, n (%)	13171 (23.3)	917 (29.5)	<0.001			
Hypertension, n (%)	44776 (79.3)	2608 (83.8)	<0.001			
Diabetes, n (%)	14041 (24.9)	954 (30.7)	<0.001			
Coronary heart disease, %	5108 (9.0)	426 (13.7)	<0.001			
Peripheral arterial disease, n (%)	3822 (6.8)	311 (10.0)	<0.001			
Regular alcohol use, n (%)	4501 (8.0)	290 (9.3)	0.007	1.3–1.75	1.51	<0.001
Smoking, n (%)	10346 (18.3)	449 (14.4)	<0.001			
Hypercholesterolemia, n (%)	30754 (54.5)	1440 (46.3)	<0.001			

*LACS* denotes lacunar syndrome, *PACS* partial anterior circulation syndrome, *TACS* total anterior circulation syndrome, *POCS* posterior circulation syndrome, *NIHSS* National Institute of Health Stoke Scale, *mRS* modified Rankin scale. Numbers are *n* and % or median and interquartile range, as indicated. *OR* denotes odds ratio, *CI* confidence interval. Multivariate analysis is continued in Table 2

The univariate comparison between the group with and without PSP showed that patients with PSP were older (79.8 vs 72.9 %, *p* < 0.001), less often functionally unimpaired before the stroke event (mRS 0 48.4 vs 70.2 %, *p* < 0.001) and had more severe strokes (median NHISS on admission 14 vs 3, *p* < 0.001). Hypertension, diabetes mellitus, and history of stroke, coronary heart disease and atrial fibrillation were significantly more common among patients with PSP (51.3 vs. 24.9 %), whereas the prevalence of hypercholesterolemia and smoking was lower.

Patients with PSP had been more often treated with thrombolysis (22 vs. 13.9 %, *p* < 0.001), continuous intravenous insulin treatment (4.2 vs 0.8 %, *p* < 0.001), insulin injections or with intravenous antihypertensive agents (12.2 vs 3.8 %, *p* < 0.001). Antiplatelets and lipid lowering agents had been less often administered to patients with PSP (50.2 vs 63.8 %, *p* < 0.001 resp. 73.3 vs 80.4 %, *p* < 0.001). Invasive procedures like endotracheal intubation (4.6 vs 0.7 %, *p* < 0.001), urinary catheter installation (79.4 vs 27.3 %, *p* < 0.001), percutaneous gastrostomy (5.6 vs. 0.5 %, *p* < 0.001) or particular insertion of nasogastric tubes (47 vs 6.8 %, *p* < 0.001) were performed in much higher frequencies in the PSP patients (Table 2).

**Table 2:** Treatment and complications

	Pneumonia at the SU					
	No	Yes	Univariate analysis		Multivariate analysis	
Treatment and complications	56447 (94.8 %)	3111 (5.2 %)	*P*	5–95 % CI	OR	*p*
iv-Thrombolytic therapy¸ n (%)	7822 (13.9)	685 (22)	<0.001			
Iv Insulin¸ n (%)	464 (0.8)	131 (4.2)	<0.001	1.22–1.98	1.55	<0.001
Iv Antihypertensive drugs, n (%)	2163 (3.8)	379 (12.2)	<0.001	1.22–1.62	1.41	<0.001
Lipid lowering drugs, n (%)	28657 (63.8)	1213 (50.2)	<0.001			
Antiplatelet drugs, n (%)	45408 (80.4)	2279 (73.3)	<0.001			
Endotracheal intubation, n (%)	400 (0.7)	143 (4.6)	<0.001			
Nasogastric tube, n (%)	3864 (6.8)	1461 (47)	<0.001	2.84–3.47	3.14	<0.001
Percutaneous gastrostomy, n (%)	286 (0.5)	175 (5.6 %	<0.001	2.02–3.14	2.52	<0.001
Urinary catheter, n (%)	15382 (27.3)	2471 (79.4)	<0.001	2.15–2.72	2.42	<0.001
Physiotherapy, n (%)	39207 (69.5)	2741 (88.1)	<0.001			
NIHSS at discharge from SU, median, interquartile range	2 (0, 5)	13 (6, 20)	<0.001			
Difference NIHSS admission – discharge, median, interquartile	1 (0, 3)	0 (−1, 3)	<0.001			
Speech therapy, n (%)	29020 (51.4)	2337 (75.1)	<0.001	1.23–1.5	1.36	<0.001
Progressive stroke, n (%)	1447 (2.6)	296 (9.5)	<0.001	1.47–2.01	1.72	<0.001
Recurrent stroke, n (%)	425 (0.8)	82 (2.6)	<0.001	1.25–2.25	1.68	<0.001
SICH, n (%)	552 (1.0)	151 (4.9)	<0.001			
Epileptic seizure, n (%)	451 (0.8)	119 (3.8)	<0.001	1.36–2.23	1.74	<0.001
Brain edema, n (%)	550 (1.0)	222 (7.1)	<0.001	1.29–1–9	1.56	<0.001
Septicemia, n (%)	141 (0.2)	87 (2.8)	<0.001	2.47–4.83	3.45	<0.001
Urinary tract infection, n (%)	2042 (3.6)	444 (14.3)	<0.001	1.1–1.42	1.25	<0.001
Heart failure, n (%)	661 (1.2)	328 (10.5)	<0.001	2.16–3.04	2.56	<0.001
Pulmonary embolism, n (%)	85 (0.2)	42 (1.4)	<0.001			
Arrhythmia, n (%)	1187 (2.1)	378 (12.2)	<0.001	1.79–2.43	2.08	<0.001

NIHSS denotes National Institute of Health Stoke Scale, SICH symptomatic intracranial hemorrhage. Numbers are n and % or median and interquartile range, as indicated. OR denotes odds ratio, CI confidence interval. Multivariate analysis is continued from Table 1

Patients with PSP were discharged from the SU significantly later (5 days, IQR 3–9 days vs. 3 days, interquartile range IQR 1–4, *p* < 0.001), had a significantly higher median NIHSS (13 vs 2, *p* < 0.001) and a lesser median decrease in NIHSS between admission and discharge from the SU than patients without PSP (0 vs 1, *p* < 0.001). In the univariate comparison, every complication listed in the registry occurred more often in stroke patients with PSP (Table 2).

For the three-month follow up data from only 24136 (40.5 %) patients were available. Among these, patients with pneumonia at the SU had a more than fivefold higher mortality than patients without PSP (42.1 vs. 7.5 %, *p* < 0.001, OR 2.99, 95%CI 2.53–3.54, multivariate model, see table in Additional file 3).

In the multiple regression model age and stroke severity were strongly associated with the occurrence of PSP. A potentially modifiable risk factor was history of atrial fibrillation and history of regular alcohol consumption (this difference between groups was very small and therefore not clinically important). Among treatment related factors treatment with intravenous insulin or antihypertensive agents, installation of urinary catheter, nasogastric tube insertion or percutaneous endoscopic gastrostomy (PEG) implantation were significantly associated with PSP. The highest OR was found for placement of nasogastric tubes (OR 3.14, %95 CI 2.84–3.41, *p* < 0.001); 47 and 7 % of patients with and without PSP had nasogastric tubes. Most of the variables found to be associated with PSP in the univariate analysis were also associated with PSP in the multivariate analysis. In our model we found also factors associated with a with lower risk of PSP: i.e. female sex, left hemispheric stroke and cryptogenic stroke etiology.

**Fig. 1:**
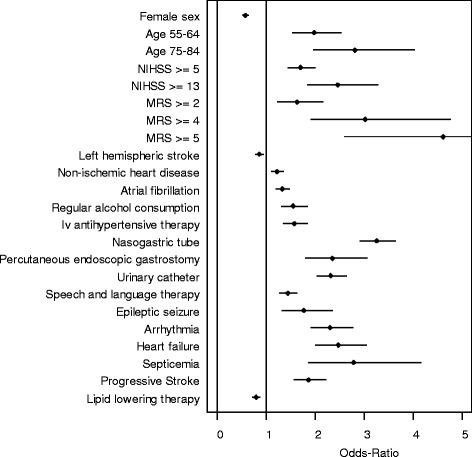
Forrest plot with odds ratios and 95 % confidence intervals. Multivariate analysis was restricted to data of patients with entries on lipid lowering therapy with bidirectional stepwise variable selection, explanatory variables are listed in Additional file 1: Table S2

In a second model restricted to the data of patients with known treatment status with lipid lowering drugs the same factors were associated with an increased risk of PSP as in the first model. Current treatment with lipid lowering drugs showed a lower risk for PSP (Fig. 1).

## Discussion

We present the so far largest register-based study on the prevalence of PSP at SUs and a comparative analysis of patients with and without PSP.

Patients with PSP were older, had more often history of regular alcohol consumption and atrial fibrillation than those without PSP. Atrial fibrillation has already been found to be an independent risk factor for in-hospital acquired pneumonia [30], it is possible that this holds true also for stroke patients. Patients with PSP had also more severe strokes on admission, a higher NIHSS at time of discharge from the SU and a smaller improvement in NIHSS between admission and discharge from the SU. This can be interpreted that PSP not only occurs more often in severe strokes but also that early recovery is impaired in patients with PSP.

Several findings are interesting for further exploration: treatment with intravenous insulin is a possible surrogate marker for severe hyperglycemia in our cohort. The occurrence of infections is increased in hyperglycemia. Elevated blood glucose can lead to impairment of immune response on one hand, while on the other hand any infection can lead to a higher blood glucose levels, so the association of PSP with blood glucose may be reciprocal. Other authors consider an altered immune status [31] in stroke patients to be causal for the development of post stroke infections, especially PSP. During the acute phase of ischemic stroke stress hormones, in particular cortisol and norepinephrine, are released and cause hyperglycemia and also lymphopenia and lymphocyte dysfunction [32–34]. The ratio between the pro-inflammatory TNF- to the anti-inflammatory IL-10 is reduced during the period of the stroke related infection [35].

Similarly, treatment with antihypertensive agents may be a surrogate for acutely elevated blood pressure. However, in the ASUR no laboratory values are collected, hence no data on the actual blood glucose level and on the actual blood pressure were available.

The association with procedures such as urinary catheter installation and tube feeding are possibly related to severe illness leading to prolonged bed rest and dysphagia. One limitation of our study is that we could not document the temporal relationship between these procedures and the onset of PSP and it is possible that they are consequences of an elevated risk for PSP due to dysphagia rather than a risk factor for PSP. Recently a retrospective study of 536 patients and a large metaanalysis [12] also found placement of nasogastric tubes to be a strong predictor of respiratory infections in the first week after stroke [36].

Interestingly, some variables showed an inverse association with the risk of PSP. A healthier life style with lower alcohol consumption and smoking habits might contribute to the lower risk in women compared with men, although these factors were controlled for in the multivariate analysis. However, it is still possible that risk factors such as alcohol consumption or smoking are underreported in men. In a community based study in the elderly male sex was a risk factor for pneumonia [37], similarly among a cohort of trauma patients in another study [38]. In addition, female sex hormones can inhibit the inflammatory reaction against bacterial antigens and lead to less severe respiratory infections [39].

The lower risk for PSP in left sided stroke that we found might be an incidental finding, but one might speculate that patients with left-sided stroke usually do not show symptoms of unawareness or deficits such as unilateral hemineglect or anosognosia. These impairing symptoms can interfere with swallowing function and contribute to dysphagia and aspiration. One previous study [40] found a significant overrepresentation of right hemispheric strokes with insula involvement in patients with PSP, the authors argued that these lesions lead to an altered immune response.

Our study found an inverse association with lipid lowering treatment and PSP. A similar finding has been reported in a single center study of thrombolysed patients [41], but not by others [42]. A metaanalysis [43] of eight studies has shown a lower risk for pneumonia in people (not restricted to stroke patients) on treatment with statins compared with those not using statins, although there was substantial heterogeneity among the studies.

Limitations of the study are that parameters can only be included from the SU-based stroke registry and not from other sources of clinical data and that standardization of measurements and data collection methods is often limited. Furthermore, due to the case control design of the data comparison, the temporal relation between clinical events or variables is often difficult to determine. The ASUR is applying several methods to assure the data quality [44] to the extent it is possible in the routine clinical settings. Further limitations are the high number (59 %) of missing values concerning the three months follow up and that PSP was diagnosed without a clearly specified diagnostic algorithm by the treating physician on the basis of general clinical rules. In addition, later occurring cases of PSP after discharge from the SU were not recorded in the registry.

The strength of our study is the large and representative patient material with full national coverage and the low differences in treatment practices in the SUs that follow national and international guidelines and SOPs. Several preventive trials with antibiotics demonstrated reduction of infection rates in patients with acute stroke but failed to improve functional outcome [45–48]. Routine application of preventive antibiotic therapy is therefore not recommended. In this light, findings from studies like ours can help to identify factors that confer higher or lower risk for PSP in stroke patients treated at SUs and accordingly guide SU care.

## Conclusion

We have shown that PSP is a frequent complication among stroke patients treated at the SU. We identified age, stroke severity, alcohol consumption and atrial fibrillation as predisposing risk factors. Components of stroke unit care and treatment procedures that are related to stroke severity, complications and dysphagia were also associated with PSP. Female sex, left hemispheric stroke, cryptogenic stroke pathogenesis and additionally, treatment with lipid lowering drugs had an inverse association with the risk of PSP. Factors both with higher and lower odds for PSP should be explored in further studies, using predetermined data collection with unbiased methodology and adequate control for confounding that are not always possible in register-based studies with limited data. Since this issue is important for stroke recovery and prognosis of patients, and for the costs associated with treatment of stroke patients, trials testing benefits of specific interventions targeted at these factors need to be designed.

## Abbreviations

ASUR, Austrian Stroke Unit Registry; CI, confidence interval; IQR, interquartile range; NIHSS, National Institute of Health stroke scale; OR, odds ratio; PSP, post stroke pneumonia; RR, relative risk; SU, stroke unit; TIA, transient ischemic attack

## Additional files

Additional file 1: Tables S1 and S2. (DOCX 381 KB) Additional file 2: “Pneumonie”, contains description of data selection process, frequencies of included variables, univariate comparisons and multivariate regression models. (DOCX 330 kb) Additional file 3: “Three months mortality”, contains results of multivariate model on study patients with complete data on three months follow up, with mortality as dependent variable. (DOCX 82 kb) 
