# Supplementary material for: Post-stroke pneumonia at the stroke unit – a registry based analysis of contributing and protective factors
Source: BMC Neurol. 2016 Jul 18;16:107. doi: 10.1186/s12883-016-0627-y (PMC4949772; doi:10.1186/s12883-016-0627-y)
Supplement: Additional file 1: — Tables S1 and S2. (DOCX 381 KB) [file 12883_2016_627_MOESM1_ESM.docx]

Table S1

Table S2
