# Supplementary material for: Post-stroke pneumonia at the stroke unit – a registry based analysis of contributing and protective factors
Source: BMC Neurol. 2016 Jul 18;16:107. doi: 10.1186/s12883-016-0627-y (PMC4949772; doi:10.1186/s12883-016-0627-y)
Supplement: Additional file 2: — “Pneumonie”, contains description of data selection process, frequencies of included variables, univariate comparisons and multivariate regression models. (DOCX 330 kb) [file 12883_2016_627_MOESM2_ESM.docx]

Pneumonie

[Leonhard Seyfan](mailto:leonhard.seyfang@goeg.at)g

6. März 2015

Inhaltsverzeichnis

| 1 | [Date](#page2)n | | 2 |
| --- | --- | --- | --- |
|  | [1.](#page2)1 | [Filterung der Daten](#page2) . . . . . . . . . . . . . . . . . . . . . . . . . . . . . . . . . . . . | 2 |
|  | 1.2 | Zusatzvariablen . . . . . . . . . . . . . . . . . . . . . . . . . . . . . . . . . . . . . . | 3 |
| 2 | [Basisdate](#page4)n | | 4 |
| 3 | [Model](#page12)l | | [1](#page12)2 |
|  | 3.1 | Variablen . . . . . . . . . . . . . . . . . . . . . . . . . . . . . . . . . . . . . . . . . . | 12 |
|  | 3.2 | Resultierendes Modell . . . . . . . . . . . . . . . . . . . . . . . . . . . . . . . . . . | 14 |
|  | 3.3 | Resultierendes Modell 2 . . . . . . . . . . . . . . . . . . . . . . . . . . . . . . . . . | 19 |

| 1 DATEN | 2 |
| --- | --- |
|  |  |

1 Daten

1.1 Filterung der Daten

Ausgewertet werden Daten aus dem Stroke-Unit-Register, Download vom 03. 12. 2013 (N = 98 705), die die folgenden Kriterien erfüllen:

- *Akuter Schlaganfall* (i2006) = *Ja*

N = 80 909

- *Syndrom* (i7001) = *Ischämie*

N = 71 312

- Vorläufige Klassifikation: *Ischämie* (i16008) = *Ja* (falls eingegeben) N = 68 583
- *Alter* > 0 N = 68 138
- *Geschlecht* (i2005) ist eingegeben N = 68 138
- *mRS vor Insult* (i6001) ist eingegeben N = 67 827
- *NIHSS bei Aufnahme* (i4020) ist eingegeben N = 67 626
- *Risikofaktoren* (i9001 - i9010) sind eingegeben N = 62 595
- *Entlassungsdatum* (i11001) ist eingegeben N = 61 735
- *Lokalisation* (i7005) ist eingegeben N = 61 734
- *Klinisches Syndrom* (i7010) ist eingegeben N = 61 734
- *mRS bei Aufnahme* (i6002) ist eingegeben N = 61 734
- *Lyse i.v.* (i10001) ist eingegeben N = 61 543
- Die Sekundärprophylaxen *ASS* (i17001), *Clopidogrel* (i17002), *ASS & DIP* (i17003), *Heparin sc.* (i17004), *Dicumarine* (i17005), *PTA* (i17006) und *CEA* (i17007) sind ein-gegeben.

N = 59 562

- *Pneumonie* (i15011) ist eingegeben 59 558

| 1 DATEN | 3 |
| --- | --- |
|  |  |

1.2 Zusatzvariablen

8

^>^Unbekannt

>

>

>

>

>

>

>

>

^>^Kardiogen Embolisch

>

>

>

>

>

>

>

>

>

>

>

>

>

>

>

^>^Makroangiopathie

>

>

>

>

>

>

>

>

>

>

>

>

>

>

>

>

>

<

Ätiologie =

^>^Mikroangiopathie

>

>

>

>

>

>

>

>

>

>

>

>

>

>

>

>

>

>

>

>

>

>

>

>

>

^>^Andere

>

>

>

>

>

>

>

>

>

>

>

>

>

>

>

^>^Unbekannt

:

8

<Ja **wenn**

Thrombolyse =

^:^Nein **wenn**

8

^>^Keines/Low

>

>

>

>

>

>

>

>

>

>

>

>

>

<

Heparin (Therapie) = ^Medium^

>

>

>

>

>

>

>

>

^>^High/iv

>

>

>

>

>

:

**wenn** Ätiopathogenese derzeit unbekannt = Ja

**wenn** Ätiopathogenese derzeit unbekannt = Nein **und**

Thromboembolie aus kard. Quelle = Wahrscheinlich

**wenn** Ätiopathogenese derzeit unbekannt = Nein **und**

Thromboembolie aus kard. Quelle 6= Wahrscheinlich **und** Makroangiopathie =

Wahrscheinlich

(1)

**wenn** Ätiopathogenese derzeit unbekannt = Nein **und**

Thromboembolie aus kard. Quelle 6= Wahrscheinlich **und** Makroangiopathie 6= Wahrscheinlich **und** Mikroangiopathie = Ja

**wenn** keine der obigen Definition zutrifft **und** (Hämodynamisch = Ja **oder** Koagulopathie = Ja **oder** Arterielle Dissektion= Ja)

**sonst**

Lyse i.v. und Lyse i.a. sind eingegeben **und**

(Lyse i.v. = Ja **oder** Lyse i.a. = Ja) (2) Lyse i.v. = Nein **und** Lyse i.a. = Nein

| **wenn** Heparin subkutan (i10004) = | | |  |  |
| --- | --- | --- | --- | --- |
|  | Keines **oder** Heparin subkutan | | = |  |
|  |  |  |  |  |
|  | Low **und** Heparin i.v. (i10003) | | |  |
|  | = | Nein |  |  |
| **wenn** |  |  |  |  |
|  | Heparin subkutan = | | (3) |  |
|  | Medium (75-150E) **und** | |  |  |
|  | Heparin i.v. = Nein | |  |  |
| **wenn** | Heparin subkutan = | |  |  |

High (*>*150E) **oder** Heparin i.v. = Ja

| 2 | BASISDATEN |  |  |  |  |  |  | 4 | |  |  |
| --- | --- | --- | --- | --- | --- | --- | --- | --- | --- | --- | --- |
|  |  |  |  |  |  |  |  |  |  |  |  |
| 2 | Basisdaten |  |  |  |  |  |  |  |  |  |  |
|  |  |  |  |  |  |  |  |  | |  |  |
|  |  | Nein |  |  | Ja |  |  | P-Wert | |  |  |
| N |  | 56447 | |  | 3111 | | |  |  |  |  |
|  | |  | |  |  | | |  | |  |  |
| Geschlecht Männlich, N (%) | | 29926 | | (53) | 1654 (53.2) | | | 0.870 | |  |  |
| Alter, Med. (*Q*_0.25_, *Q*_0.75_) | | 72.9 (62.8, 81.3) | | | 79.8 (72, 85.1) | | | *<*0.001^†^ | | |  |
| MRS vor Insult 0, N (%) | | 39601 | | (70.2) | 1507 (48.4) | | | *<*0.001^‡^ | | |  |
| MRS vor Insult 1, N (%) | | 6348 | (11.2) | | 467 | | (15) | *<*0.001^‡^ | | |  |
| MRS vor Insult 2, N (%) | | 3706 | (6.6) | | 330 | | (10.6) | *<*0.001^‡^ | | |  |
| MRS vor Insult 3, N (%) | | 3982 | (7.1) | | 456 | | (14.7) | *<*0.001^‡^ | | |  |
| MRS vor Insult 4, N (%) | | 2477 | (4.4) | | 303 | | (9.7) | *<*0.001^‡^ | | |  |
| MRS vor Insult 5, N (%) | | 333 (0.6) | | | 48 | (1.5) | | *<*0.001^‡^ | | |  |
| NIHSS bei Aufnahme, Med. (*Q*_0.25_, *Q*_0.75_) | | 3 (1, 7) | | | 14 | (8, 20) | | *<*0.001^†^ | | |  |
| MRS bei Aufnahme 0, N (%) | | 5848 | (10.4) | | 22 | (0.7) | | *<*0.001^‡^ | |  |  |
| MRS bei Aufnahme 1, N (%) | | 10999 | | (19.5) | 64 | (2.1) | | *<*0.001^‡^ | | |  |
| MRS bei Aufnahme 2, N (%) | | 7977 | (14.1) | | 92 | (3) | | *<*0.001^‡^ | | |  |
| MRS bei Aufnahme 3, N (%) | | 8634 | (15.3) | | 169 | | (5.4) | *<*0.001^‡^ | | |  |
| MRS bei Aufnahme 4, N (%) | | 16104 | | (28.5) | 1072 (34.5) | | | *<*0.001^‡^ | | |  |
| MRS bei Aufnahme 5, N (%) | | 6885 | (12.2) | | 1692 (54.4) | | | *<*0.001^‡^ | | |  |
| Zutransport KH Rettung mit Notarzt, N (%) | | 2239 | (30.5) | | 162 | | (39.1) | *<*0.001^‡^ | |  |  |
| Zutransport KH Rettung ohne Notarzt, N (%) | | 4753 | (64.9) | | 224 | | (54.1) | *<*0.001^‡^ | | |  |
| Zutransport KH Rettungshubschrauber, N (%) | | 337 (4.6) | | | 28 | (6.8) | | 0.043^‡^ | | |  |
| Zutransport direkt Rettung mit Notarzt, N (%) | | 11051 | | (22.6) | 1049 (39.2) | | | *<*0.001^‡^ | | |  |
| Zutransport direkt Rettung ohne Notarzt, N (%) | | 26658 | | (54.6) | 1303 (48.7) | | | *<*0.001^‡^ | | |  |
| Zutransport direkt Rettungshubschrauber, N (%) | | 1488 | (3) | | 153 | | (5.7) | *<*0.001^‡^ | | |  |
| Zutransport direkt Privat, N (%) | | 9138 | (18.7) | | 112 | | (4.2) | *<*0.001^‡^ | | |  |
| Zutransport direkt Anderer, N (%) | | 513 (1.1) | | | 61 | (2.3) | | *<*0.001^‡^ | | |  |
| Zutransport intern Ambulanz, N (%) | | 10570 | | (18.7) | 406 | | (13.1) | *<*0.001^‡^ | | |  |
| Zutransport intern Andere Fachabteilung, N (%) | | 3094 | (5.5) | | 276 | | (8.9) | *<*0.001^‡^ | | |  |
| Zutransport intern Direkt an die SU, N (%) | | 18479 | | (32.7) | 1069 (34.4) | | | 0.060^‡^ | | |  |
| Zutransport intern Notaufnahme, N (%) | | 22694 | | (40.2) | 1261 (40.5) | | | 0.715^‡^ | | |  |
| Zutransport intern Eigene Fachabteilung, N (%) | | 1610 | (2.9) | | 99 | (3.2) | | 0.283^‡^ | |  |  |

Tabelle 1: Aufnahmedaten 1: Geschlecht, Alter, Schweregrad, Zutransport

Chi-Quadrat-Unabhängigkeitstest für den *m g* - Fall

^†^Kruskal-Wallis-Test

^‡^Chi-Quadrat-Unabhängigkeitstest für den 2 *g* - Fall

| 2 BASISDATEN |  |  |  |  |  |  |  | 5 |  |  |  |
| --- | --- | --- | --- | --- | --- | --- | --- | --- | --- | --- | --- |
|  |  |  |  |  |  |  |  |  |  |  |  |
|  |  |  |  |  |  |  |  |  | |  |  |
|  | Nein |  |  | Ja |  |  |  | P-Wert | |  |  |
| N | 56447 | |  | 3111 | | |  |  |  |  |  |
|  |  | |  |  | | |  |  | |  |  |
| Lokalisation Linke Großhirnhemisphäre, N (%) | 25692 | | (45.5) | 1446 | | | (46.5) | 0.293^‡^ | |  |  |
| Lokalisation Rechte Großhirnhemisphäre, N (%) | 19853 | | (35.2) | 1250 | | | (40.2) | *<*0.001^‡^ | | |  |
| Lokalisation Beide Großhirnhemisphären, N (%) | 987 (1.7) | | | 67 | (2.2) | | | 0.095^‡^ | | |  |
| Lokalisation Vorwiegend Hirnstamm, N (%) | 7080 | (12.5) | | 269 | | (8.6) | | *<*0.001^‡^ | | |  |
| Lokalisation Vorwiegend Kleinhirn, N (%) | 2835 | (5) | | 79 | (2.5) | | | *<*0.001^‡^ | | |  |
| Klinisches Syndrom LACS, N (%) | 17592 | | (31.2) | 362 | | (11.6) | | *<*0.001^‡^ | |  |  |
| Klinisches Syndrom TACS, N (%) | 5678 | (10.1) | | 1069 | | | (34.4) | *<*0.001^‡^ | | |  |
| Klinisches Syndrom PACS, N (%) | 21627 | | (38.3) | 1214 | | | (39) | 0.428^‡^ | | |  |
| Klinisches Syndrom POCS, N (%) | 9555 | (16.9) | | 386 | | (12.4) | | *<*0.001^‡^ | | |  |
| Klinisches Syndrom Anderes, N (%) | 1995 | (3.5) | | 80 | (2.6) | | | 0.004^‡^ | | |  |
| Ereignis aufgetreten Im Wachzustand, N (%) | 40565 | | (71.9) | 1832 | | | (58.9) | *<*0.001^‡^ | |  |  |
| Ereignis aufgetreten Im Schlaf, N (%) | 8212 | (14.5) | | 522 | | (16.8) | | *<*0.001^‡^ | | |  |
| Ereignis aufgetreten Unbekannt, N (%) | 7670 | (13.6) | | 757 | | (24.3) | | *<*0.001^‡^ | | |  |
| ODT, Med. (*Q*_0.25_, *Q*_0.75_) | 120 (61, 280) | | | 90 | (57, 156) | | | *<*0.001^†^ | |  |  |
| Zeit 1. Bildgebung, Med. (*Q*_0.25_, *Q*_0.75_) | 30 (15, 60) | | | 25 | (15, 45) | | | *<*0.001^†^ | | |  |
| Zeit hirnzuf. Gefäße, Med. (*Q*_0.25_, *Q*_0.75_) | 152 (50, 1155) | | | 210 | | (48, 1385.5) | | *<*0.001^†^ | | |  |
| CCT Ja, N (%) | 46684 | | (83.1) | 2688 | | | (86.6) | *<*0.001^‡^ | |  |  |
| MRI Ja, N (%) | 9828 | (17.5) | | 317 | | (10.2) | | *<*0.001^‡^ | | |  |
| Duplex Ja, N (%) | 42715 | | (76.1) | 2217 | | | (71.4) | *<*0.001^‡^ | | |  |
| Doppler Ja, N (%) | 37274 | | (68.5) | 1979 | | | (65.3) | *<*0.001^‡^ | | |  |
| MR-Angio Ja, N (%) | 7252 | (12.9) | | 275 | | (8.9) | | *<*0.001^‡^ | | |  |
| Standardmonitoring Ja, N (%) | 55043 | | (98) | 3065 | | | (98.7) | 0.005^‡^ | | |  |
| RR blutig Ja, N (%) | 964 (1.7) | | | 194 | | (6.2) | | *<*0.001^‡^ | | |  |
| Hirndrucksonde Ja, N (%) | 80 (0.1) | | | 6 (0.2) | | | | 0.461^¶^ | |  |  |
| TCD-Monitoring Ja, N (%) | 368 (0.7) | | | 15 | (0.5) | | | 0.244^‡^ | | |  |
| ZVD Ja, N (%) | 150 (0.3) | | | 80 | (2.6) | | | *<*0.001^‡^ | | |  |
| EEG Ja, N (%) | 1779 | (3.2) | | 103 | | (3.3) | | 0.643^‡^ | |  |  |

Tabelle 2: Aufnahmedaten 2

^†^Kruskal-Wallis-Test

^‡^Chi-Quadrat-Unabhängigkeitstest für den 2 *g* - Fall ^¶^Exakter Test nach Fisher für den 2 *g* - Fall

| 2 BASISDATEN | |  |  |  |  | 6 | |  |
| --- | --- | --- | --- | --- | --- | --- | --- | --- |
|  |  |  |  |  |  |  |  |  |
|  |  |  |  |  |  |  |  |  |
|  |  | Nein | Ja |  |  | P-Wert |  |  |
|  | N | 56447 | 3111 | |  |  |  |  |
|  |  |  |  | |  |  |  |  |
|  | Hypertonie Ja, N (%) | 44776 (79.3) | 2608 | | (83.8) | *<*0.001^‡^ |  |  |
|  | Diabetes mellitus Ja, N (%) | 14041 (24.9) | 954 | (30.7) | | *<*0.001^‡^ | |  |
|  | Vorinsult Ja, N (%) | 13171 (23.3) | 917 | (29.5) | | *<*0.001^‡^ | |  |
|  | Herzinfarkt Ja, N (%) | 5108 (9) | 426 | (13.7) | | *<*0.001^‡^ | |  |
|  | Hypercholesterinämie Ja, N (%) | 30754 (54.5) | 1440 | | (46.3) | *<*0.001^‡^ | |  |
|  | Vorhofflimmern Ja, N (%) | 14031 (24.9) | 1596 | | (51.3) | *<*0.001^‡^ | |  |
|  | Andere kardiale Erkrankung Ja, N (%) | 12178 (21.6) | 1057 | | (34) | *<*0.001^‡^ | |  |
|  | PAVK Ja, N (%) | 3822 (6.8) | 311 | (10) | | *<*0.001^‡^ | |  |
|  | Nikotin Ja, N (%) | 10346 (18.3) | 449 | (14.4) | | *<*0.001^‡^ | |  |
|  | Alkohol regelmäßig Ja, N (%) | 4501 (8) | 290 | (9.3) | | 0.007^‡^ | |  |
|  | Alkoholintoxikation Ja, N (%) | 282 (0.5) | 22 (0.7) | | | 0.114^‡^ |  |  |

Tabelle 3: Risikofaktoren

|  | Nein |  | Ja |  |  | P-Wert | |  |
| --- | --- | --- | --- | --- | --- | --- | --- | --- |
| N | 56447 | | 3111 | |  |  |  |  |
|  |  |  |  |  | |  |  |  |
| Lyse i.v. Ja, N (%) | 7822 | (13.9) | 685 | (22) | | *<*0.001^‡^ |  |  |
| Lyse i.a. Ja, N (%) | 457 (0.8) | | 83 (2.7) | | | *<*0.001^‡^ | |  |
| Heparin i.v. Ja, N (%) | 2793 | (4.9) | 124 | (4) | | 0.015^‡^ | |  |
| Heparin subkutan Keines, N (%) | 7374 | (13.1) | 291 | (9.4) | | *<*0.001^‡^ | |  |
| Heparin subkutan Low (*<*75E), N (%) | 38912 (68.9) | | 2195 | | (70.6) | 0.057^‡^ | |  |
| Heparin subkutan Medium (75-150E), N (%) | 8342 | (14.8) | 494 | (15.9) | | 0.093^‡^ | |  |
| Heparin subkutan High (>150E), N (%) | 1819 | (3.2) | 131 | (4.2) | | 0.003^‡^ | |  |
| Thrombozytenaggregationshemmer Ja, N (%) | 45408 (80.4) | | 2279 | | (73.3) | *<*0.001^‡^ |  |  |
| Andere zerebrovasculäre Akutmedikation Ja, N (%) | 8007 | (14.2) | 544 | (17.5) | | *<*0.001^‡^ | |  |
| Assistierte oder kontrollierte Beatmung Ja, N (%) | 602 (1.1) | | 149 | (4.8) | | *<*0.001^‡^ | |  |
| Kontinuierliche i.v. Therapie Ja, N (%) | 6329 | (11.2) | 865 | (27.8) | | *<*0.001^‡^ | |  |
| Insulin Ja, N (%) | 464 (0.8) | | 131 | (4.2) | | *<*0.001^‡^ | |  |
| Dopamin Ja, N (%) | 51 (0.1) | | 26 (0.8) | | | *<*0.001^‡^ | |  |
| Antihypertensiva Ja, N (%) | 2163 | (3.8) | 379 | (12.2) | | *<*0.001^‡^ | |  |
| Andere Therapie Ja, N (%) | 4613 | (8.2) | 602 | (19.4) | | *<*0.001^‡^ | |  |
| Intubation Ja, N (%) | 400 (0.7) | | 143 | (4.6) | | *<*0.001^‡^ | |  |
| Nasogastrische Sonde Ja, N (%) | 3864 | (6.8) | 1461 | | (47) | *<*0.001^‡^ | |  |
| Perkutane Gastrostomie Ja, N (%) | 286 (0.5) | | 175 | (5.6) | | *<*0.001^‡^ | |  |
| Blasenkatheter Ja, N (%) | 15382 (27.3) | | 2471 | | (79.4) | *<*0.001^‡^ | |  |
| Op. Dekompression Ja, N (%) | 155 (0.3) | | 16 (0.5) | | | 0.015^‡^ | |  |
| Klinische Prüfung Ja, N (%) | 2348 | (4.2) | 143 | (4.6) | | 0.236^‡^ | |  |
| Physiotherapie Ja, N (%) | 39207 (69.5) | | 2741 | | (88.1) | *<*0.001^‡^ | |  |
| Ergotherapie Ja, N (%) | 31661 (56.1) | | 2049 | | (65.9) | *<*0.001^‡^ | |  |
| Logopädie Ja, N (%) | 29020 (51.4) | | 2337 | | (75.1) | *<*0.001^‡^ | |  |
| DNT, Med. (*Q*_0.25_, *Q*_0.75_) | 47 (30, 70) | | 45 (30, 67) | | | 0.606^†^ |  |  |

Tabelle 4: Therapie

| 2 BASISDATEN |  |  |  |  |  |  | 7 |  |  |
| --- | --- | --- | --- | --- | --- | --- | --- | --- | --- |
|  |  |  |  |  | |  |  |  |  |
|  |  |  |  |  | |  |  | |  |
|  | Nein |  |  | Ja | |  | P-Wert | |  |
| N | 56447 | |  | 3111 | | |  |  |  |
|  |  | | |  |  | |  | |  |
| Liegedauer, Med. (*Q*_0.25_, *Q*_0.75_) | 3 (1, 4) | | | 5 | (3, 9) | | *<*0.001^†^ | |  |
| Entlassungsart Nach Hause, N (%) | 5985 | (10.6) | | 43 | | (1.4) | *<*0.001^‡^ | |  |
| Entlassungsart Neurolog. Akutbett, N (%) | 32346 | | (57.3) | 1357 (43.6) | | | *<*0.001^‡^ | |  |
| Entlassungsart Neurolog. Phase B, N (%) | 5071 | (9) | | 759 (24.4) | | | *<*0.001^‡^ | |  |
| Entlassungsart Neurolog. Phase C im Haus, N (%) | 6998 | (12.4) | | 335 (10.8) | | | 0.007^‡^ | |  |
| Entlassungsart Neurolog. Phase C außer Haus, N (%) | 939 (1.7) | | | 84 | | (2.7) | *<*0.001^‡^ | |  |
| Entlassungsart Tagesklinik im Haus, N (%) | 16 (0) | |  | 0 | (0) | | 1.000^¶^ | |  |
| Entlassungsart Tagesklinik außer Haus, N (%) | 7 (0) |  |  | 0 | (0) | | 0.790^¶^ | |  |
| Entlassungsart Neurolog. Phase D außer Haus, N (%) | 68 (0.1) | | | 4 | (0.1) | |  |  |  |
| Entlassungsart Geriatrische Abteilung im Haus, N (%) | 758 (1.3) | | | 78 | | (2.5) | *<*0.001^¶^ | |  |
| Entlassungsart Geriatrische Abteilung außer Haus, N (%) | 95 (0.2) | | | 5 | (0.2) | | 1.000^¶^ | |  |
| Entlassungsart Neurochirurg. Abteilung im Haus, N (%) | 134 (0.2) | | | 3 | (0.1) | | 0.124^¶^ | |  |
| Entlassungsart Neurochirurg. Abteilung außer Haus, N (%) | 183 (0.3) | | | 6 | (0.2) | | 0.251^¶^ | |  |
| Entlassungsart Andere Fachabteilung im Haus, N (%) | 1257 | (2.2) | | 67 | | (2.2) | 0.851^¶^ | |  |
| Entlassungsart Andere Fachabteilung außer Haus, N (%) | 1531 | (2.7) | | 114 (3.7) | | | 0.002^¶^ | |  |
| Entlassungsart Intensivstation, N (%) | 566 (1) | | | 198 (6.4) | | | *<*0.001^¶^ | |  |
| Entlassungsart Pflegeheim, N (%) | 358 (0.6) | | | 29 | | (0.9) | 0.051^¶^ | |  |
| Entlassungsart Altersheim, N (%) | 83 (0.1) | | | 3 | (0.1) | | 0.630^¶^ | |  |
| Entlassungsart Verstorben, N (%) | 47 (0.1) | | | 25 | | (0.8) | *<*0.001^¶^ | |  |
| NIHSS bei Entlassung, Med. (*Q*_0.25_, *Q*_0.75_) | 2 (0, 5) | | | 13 | | (6, 20) | *<*0.001^†^ | |  |
| MRS bei Entlassung 0, N (%) | 13808 | | (24.6) | 47 | | (1.5) | *<*0.001^¶^ | |  |
| MRS bei Entlassung 1, N (%) | 12222 | | (21.8) | 72 | | (2.3) | *<*0.001^¶^ | |  |
| MRS bei Entlassung 2, N (%) | 6699 | (11.9) | | 81 | | (2.6) | *<*0.001^¶^ | |  |
| MRS bei Entlassung 3, N (%) | 7249 | (12.9) | | 147 (4.7) | | | *<*0.001^¶^ | |  |
| MRS bei Entlassung 4, N (%) | 10654 | | (19) | 921 (29.7) | | | *<*0.001^¶^ | |  |
| MRS bei Entlassung 5, N (%) | 5459 | (9.7) | | 1799 (58.1) | | | *<*0.001^¶^ | |  |
| MRS bei Entlassung 6, N (%) | 60 (0.1) | | | 30 | | (1) | *<*0.001^¶^ | |  |
| Verbesserung NIHSS, Med. (*Q*_0.25_, *Q*_0.75_) | 1 (0, 3) | | | 0 | (-1, 3) | | *<*0.001^†^ | |  |
| Lipidsenker Ja, N (%) | 28657 | | (63.8) | 1213 (50.2) *<*0.001^‡^ | | | | |  |

Tabelle 5: Entlassung

^†^Kruskal-Wallis-Test

- Chi-Quadrat-Unabhängigkeitstest für den 2 *g* - Fall

^†^Kruskal-Wallis-Test

- Chi-Quadrat-Unabhängigkeitstest für den 2 *g* - Fall ^¶^Exakter Test nach Fisher für den 2 *g* - Fall

| 2 BASISDATEN | |  |  |  |  |  | 8 | |  |
| --- | --- | --- | --- | --- | --- | --- | --- | --- | --- |
|  |  |  | |  |  |  |  |  |  |
|  |  |  | |  |  |  |  |  |  |
|  |  | Nein | | Ja |  |  | P-Wert |  |  |
|  | N | 56447 | | 3111 | | |  |  |  |
|  |  |  |  |  |  | |  |  |  |
|  | Reinsult Ja, N (%) | 425 | (0.8) | 82 | (2.6) | | *<*0.001^¶^ |  |  |
|  | Klin. Relevante Einblutung Ja, N (%) | 552 | (1) | 151 | | (4.9) | *<*0.001^¶^ |  |  |
|  | Hirnödem Ja, N (%) | 550 | (1) | 222 | | (7.1) | *<*0.001^¶^ |  |  |
|  | Epil. Anfall Ja, N (%) | 451 | (0.8) | 119 | | (3.8) | *<*0.001^¶^ |  |  |
|  | Hydrocephalus Ja, N (%) | 47 (0.1) | | 10 | (0.3) | | *<*0.001^¶^ |  |  |
|  | Kardiale Arrythmien Ja, N (%) | 1187 (2.1) | | 378 | | (12.2) | *<*0.001^¶^ |  |  |
|  | Herz-Kreislaufdekompensation Ja, N (%) | 661 | (1.2) | 328 | | (10.5) | *<*0.001^¶^ |  |  |
|  | Pulmonalembolie Ja, N (%) | 85 (0.2) | | 42 | (1.4) | | *<*0.001^¶^ |  |  |
|  | Sepsis Ja, N (%) | 141 | (0.2) | 87 | (2.8) | | *<*0.001^¶^ |  |  |
|  | Harnwegsinfekt Ja, N (%) | 2042 (3.6) | | 444 | | (14.3) | *<*0.001^¶^ |  |  |
|  | Extrazerebrale Blutung Ja, N (%) | 168 | (0.3) | 32 | (1) | | *<*0.001^¶^ |  |  |
|  | Tiefe Beinvenenthrombose Ja, N (%) | 65 (0.1) | | 23 | (0.7) | | *<*0.001^¶^ |  |  |
|  | Progressive Stroke Ja, N (%) | 1447 (2.6) | | 296 | | (9.5) | *<*0.001^¶^ |  |  |
|  | Herzinfarkt (K) Ja, N (%) | 335 | (0.6) | 63 | (2) | | *<*0.001^¶^ |  |  |

Tabelle 6: Komplikationen

^¶^Exakter Test nach Fisher für den 2 *g* - Fall

| 2 BASISDATEN |  | | | |  |  | | | |  | | 9 |  |  |
| --- | --- | --- | --- | --- | --- | --- | --- | --- | --- | --- | --- | --- | --- | --- |
|  |  | | | |  |  | | | |  | |  |  |  |
|  |  | | | |  |  | | | |  | |  | |  |
|  | Nein | | | |  | Ja | | | |  | | P-Wert | |  |
| N | 56447 | | | |  | 3111 | | | |  | |  |  |  |
|  |  | | | |  |  | | | |  | |  |  |  |
| Ischämie Ja, N (%) | 56335 | | | | (100) | 3106 | | | | (100) | |  |  |  |
| Art der Ischämie TIA, N (%) | 13878 | | | | (24.7) | 96 (3.1) | | | | | | *<*0.001^‡^ | |  |
| Art der Ischämie PRIND, N (%) | 7037 (12.5) | | | | | 85 (2.7) | | | | | | *<*0.001^‡^ | |  |
| Art der Ischämie Kompletter Hirninfarkt, N (%) | 35365 | | | | (62.8) | 2922 | | | | (94.2) | | *<*0.001^‡^ | |  |
| Mikroangiopathie Ja, N (%) | 25317 | | | | (45.1) | 1105 | | | | (35.8) | | *<*0.001^‡^ | |  |
| Thromboembolie aus kard. Quelle Wahrscheinlich, N (%) 15327 | | | | | (27.3) | 1533 | | | | (49.6) | | *<*0.001^‡^ | |  |
| Thromboembolie aus kard. Quelle Möglich, N (%) | | 11585 (20.7) | | | | 625 | | | (20.2) | | 0.577^‡^ | | |  |
| Makroangiopathie Wahrscheinlich, N (%) | | 9516 | | (17) | | 601 | | | (19.5) | | *<*0.001^‡^ | | |  |
| Makroangiopathie Möglich, N (%) | | 9420 | | (16.8) | | 515 | | | (16.7) | | 0.863^‡^ | | |  |
| Hämodynamisch Ja, N (%) | | 3932 | | (7) | | 218 | | | (7.1) | | 0.918^‡^ | | |  |
| Koagulopathie Ja, N (%) | | 0 (0) | |  | | 0 | (0) | | | | *<*0.001^‡^ | | |  |
| Arterielle Dissektion Ja, N (%) | | 788 (1.4) | | | | 21 | | (0.7) | | |  |  |  |  |
| Ätiopathogenese derzeit unbekannt Ja, N (%) | | 14468 (25.8) | | | | 503 | | | (16.3) | | *<*0.001^‡^ | | |  |
| Ätiologie Mikroangiopathie, N (%) | | 15173 (26.9) | | | | 487 | | | (15.7) | | *<*0.001^‡^ | | |  |
| Ätiologie Makroangiopathie, N (%) | | 7255 | | (12.9) | | 413 | | | (13.3) | | 0.493^‡^ | | |  |
| Ätiologie Kardiogen Embolisch, N (%) | | 13649 (24.2) | | | | 1439 (46.3) *<*0.001^‡^ | | | | | | | |  |
| Ätiologie Andere, N (%) | | 1401 | | (2.5) | | 56 | | (1.8) | | | 0.017^‡^ | | |  |
| Ätiologie Unbekannt, N (%) | | 18969 (33.6) | | | | 716 | | | (23) | | *<*0.001^‡^ | | |  |
| Intrazerebrale Blutung Ja, N (%) | | 245 (0.4) | | | | 49 | | (1.6) | | | *<*0.001^‡^ | | |  |
| Hypertonie Ja, N (%) | | 57 | (35.2) | | | 18 | | (56.2) | | | 0.025^‡^ | | |  |
| Gefäßmalformation Ja, N (%) | | 7 (4.3) | | | | 1 | (3.1) | | | | 1.000^¶^ | | |  |
| Amyloidangiopathie Ja, N (%) | | 9 (5.6) | | | | 2 | (6.2) | | | | 1.000^¶^ | | |  |
| Medikamentöse Ursache Ja, N (%) | | 65 | (40.1) | | | 9 | (28.1) | | | | 0.202^‡^ | | |  |
| Subarachnoidalblutung Ja, N (%) | | 46 | (0.1) | | | 6 | (0.2) | | | | 0.053^¶^ | | |  |
| Hirnvenenthrombose Ja, N (%) | | 44 | (0.1) | | | 2 | (0.1) | | | | 1.000^¶^ | | |  |

Tabelle 7: Vorläufige Klassifikation, Ätiologie

|  | Nein | Ja |  |  | P-Wert | |  |
| --- | --- | --- | --- | --- | --- | --- | --- |
| N | 56447 | 3111 | |  |  |  |  |
|  |  |  | |  |  |  |  |
| ASS Ja, N (%) | 31932 (56.6) | 1579 | | (50.8) | *<*0.001^‡^ |  |  |
| Clopidogrel Ja, N (%) | 11917 (21.1) | 484 | (15.6) | | *<*0.001^‡^ | |  |
| ASS & DIP Ja, N (%) | 3458 (6.1) | 103 | (3.3) | | *<*0.001^‡^ | |  |
| Heparin sc. Ja, N (%) | 29836 (52.9) | 2241 | | (72) | *<*0.001^‡^ | |  |
| Dicumarine Ja, N (%) | 4777 (8.5) | 151 | (4.9) | | *<*0.001^‡^ | |  |
| Perkut. Transluminale Angioplastie Ja, N (%) | 462 (0.8) | 25 (0.8) | | | 0.929^‡^ | |  |
| Carotis-Thrombendarteriektomie Ja, N (%) | 819 (1.5) | 22 (0.7) | | | *<*0.001^‡^ | |  |
| Heparin iv. Ja, N (%) | 1544 (2.7) | 55 (1.8) | | | 0.001^‡^ | |  |
| Lipidsenker Ja, N (%) | 28657 (63.8) | 1213 | | (50.2) | *<*0.001^‡^ | |  |
| Antihypertensiva Ja, N (%) | 34860 (77.6) | 1896 | | (78.5) | 0.327^‡^ |  |  |

Tabelle 8: Sekundärprophylaxe

^‡^Chi-Quadrat-Unabhängigkeitstest für den 2 *g* - Fall ^¶^Exakter Test nach Fisher für den 2 *g* - Fall

| 2 BASISDATEN |  |  |  |  |  |  | 10 |  |  |  |
| --- | --- | --- | --- | --- | --- | --- | --- | --- | --- | --- |
|  |  | |  |  |  |  |  |  |  |  |
|  |  | |  |  |  |  |  | |  |  |
|  | Nein | |  | Ja |  |  | P-Wert | |  |  |
| N | 56447 | | | 3111 | | |  |  |  |  |
|  |  | | |  | |  | | |  |  |
| Patientenstatus Lebend, kooperativ, N (%) | 20195 (88.5) | | | 716 | | (54.4) *<*0.001^‡^ | | |  |  |
| Patientenstatus Reinsult, N (%) | 146 | (0.6) | | 11 | (0.8) | | 0.388^‡^ | | |  |
| Patientenstatus Verstorben, N (%) | 1720 | | (7.5) | 553 | | (42.1) *<*0.001^‡^ | | | |  |
| Patientenstatus Lebend, keine weiteren Daten, N (%) | 760 | (3.3) | | 35 | (2.7) | | 0.186^‡^ | | |  |
| Todesursache Hirnödem, N (%) | 257 | (14.9) | | 81 | (14.6) | | 0.866^‡^ | |  |  |
| Todesursache Reinsult, N (%) | 189 | (11) | | 41 | (7.4) | | 0.015^‡^ | | |  |
| Todesursache Herzinfarkt, N (%) | 72 (4.2) | | | 13 | (2.4) | | 0.048^‡^ | | |  |
| Todesursache Andere kardiale Ursachen, N (%) | 236 | (13.7) | | 78 | (14.1) | | 0.820^‡^ | | |  |
| Todesursache Lungenembolie, N (%) | 39 (2.3) | | | 9 (1.6) | | | 0.363^‡^ | | |  |
| Todesursache Interkurrente andere Erkrankungen, N (%) | 335 | (19.5) | | 156 | | (28.2) *<*0.001^‡^ | | | |  |
| Todesursache Konsumierende andere Erkrankungen, N (%) | 149 | (8.7) | | 40 | (7.2) | | 0.290^‡^ | | |  |
| Todesursache Andere, N (%) | 443 | (25.8) | | 135 | | (24.4) | 0.528^‡^ | | |  |
| Rehabilitation Rehabilitation, N (%) | 7360 | | (37.9) | 406 | | (59.8) *<*0.001^‡^ | | |  |  |
| Rehabilitation Keine Rehabilitation, N (%) | 11005 (56.6) | | | 248 | | (36.5) *<*0.001^‡^ | | | |  |
| Rehabilitation Keine Information, N (%) | 1062 | | (5.5) | 25 | (3.7) | | 0.043^‡^ | | |  |
| Heimhilfe Ja, N (%) | 2477 | | (12.2) | 142 | | (19.2) | *<*0.001^‡^ | |  |  |
| Diplomierte Krankenschwester Ja, N (%) | 1156 | | (5.7) | 91 | (12.3) | | *<*0.001^‡^ | | |  |
| Essen auf Rädern Ja, N (%) | 1177 | | (5.8) | 54 | (7.3) | | 0.087^‡^ | | |  |
| Pflegegeldbezieher Ja, N (%) | 3712 | | (18.3) | 254 | | (34.5) | *<*0.001^‡^ | |  |  |
| Pflegegeldstufe, Med. (*Q*_0.25_, *Q*_0.75_) | 3 (2, 4) | | | 5 (3, 5) | | | *<*0.001^†^ | | |  |
| Wohnsituation Zuhause alleine, N (%) | 3959 | | (19.5) | 76 | (10.3) | | *<*0.001^‡^ | |  |  |
| Wohnsituation Zuhause mit Verwandten / Bekannten, N (%) | 14477 (71.2) | | | 384 | | (51.8) *<*0.001^‡^ | | | |  |
| Wohnsituation Pensionistenheim, N (%) | 393 | (1.9) | | 20 | (2.7) | | 0.139^‡^ | | |  |
| Wohnsituation Pflegeheim, N (%) | 1290 | | (6.3) | 215 | | (29) | *<*0.001^‡^ | |  |  |
| Wohnsituation Krankenhaus, N (%) | 227 | (1.1) | | 46 | (6.2) | | *<*0.001^‡^ | | |  |
| Regelmäßig Dicumarine Ja, N (%) | 4442 | | (21.9) | 146 | | (19.8) | 0.184^‡^ | | |  |
| Regelmäßig Lipidsenker Ja, N (%) | 12122 (59.8) | | | 284 | | (38.6) *<*0.001^‡^ | | | |  |
| Regelmäßig Antidepressiva Ja, N (%) | 5172 | | (25.5) | 313 | | (42.5) *<*0.001^‡^ | | | |  |
| Regelmäßig Antihypertensiva Ja, N (%) | 14719 (72.6) | | | 512 | | (69.6) | 0.074^‡^ | | |  |
| Gesamtsumme Barthel Index, Med. (*Q*_0.25_, *Q*_0.75_) | 90 (55, 100) | | | 10 | (0, 30) | | *<*0.001^†^ | |  |  |

Tabelle 9: Follow-Up

^†^Kruskal-Wallis-Test

^‡^Chi-Quadrat-Unabhängigkeitstest für den 2 *g* - Fall

| 2 BASISDATEN | |  |  | 11 | | |  |
| --- | --- | --- | --- | --- | --- | --- | --- |
|  |  |  |  |  |  |  |  |
|  |  |  |  |  |  |  |  |
|  |  | Nein | Ja | Prozent |  |  |  |
|  | WA | 1237 | 93 | 7.0 | |  |  |
|  | ZO | 1094 | 125 | 10.3 | |  |  |
|  | XE | 390 | 40 | 9.3 | |  |  |
|  | RE | 2020 | 88 | 4.2 | |  |  |
|  | WI | 856 | 39 | 4.4 | |  |  |
|  | SU | 1253 | 23 | 1.8 | |  |  |
|  | RA | 3755 | 199 | 5.0 | |  |  |
|  | KE | 2377 | 50 | 2.1 | |  |  |
|  | MI | 801 | 53 | 6.2 | |  |  |
|  | TI | 1690 | 88 | 4.9 | |  |  |
|  | HI | 2879 | 139 | 4.6 | |  |  |
|  | FI | 1659 | 59 | 3.4 | |  |  |
|  | HU | 1918 | 74 | 3.7 | |  |  |
|  | BU | 384 | 36 | 8.6 | |  |  |
|  | LO | 1163 | 65 | 5.3 | |  |  |
|  | HO | 152 | 5 | 3.2 | |  |  |
|  | LA | 3833 | 174 | 4.3 | |  |  |
|  | RI | 389 | 4 | 1.0 | |  |  |
|  | JO | 467 | 34 | 6.8 | |  |  |
|  | ZA | 2584 | 247 | 8.7 | |  |  |
|  | ZU | 2654 | 148 | 5.3 | |  |  |
|  | BA | 878 | 79 | 8.3 | |  |  |
|  | TO | 4539 | 201 | 4.2 | |  |  |
|  | JA | 423 | 49 | 10.4 | |  |  |
|  | TA | 2712 | 79 | 2.8 | |  |  |
|  | LI | 663 | 27 | 3.9 | |  |  |
|  | WE | 2141 | 99 | 4.4 | |  |  |
|  | SI | 418 | 3 | 0.7 | |  |  |
|  | TU | 791 | 44 | 5.3 | |  |  |
|  | FU | 1976 | 134 | 6.4 | |  |  |
|  | BE | 1347 | 104 | 7.2 | |  |  |
|  | MO | 1363 | 50 | 3.5 | |  |  |
|  | RO | 1544 | 130 | 7.8 | |  |  |
|  | FE | 1342 | 132 | 9.0 | |  |  |
|  | SA | 1319 | 86 | 6.1 | |  |  |
|  | XO | 1436 | 111 | 7.2 | |  |  |
|  |  |  |  |  |  |  |  |

Tabelle 10: Pneumonie nach Zentrum

| 3 MODELL | 12 |
| --- | --- |
|  |  |

3 Modell

3.1 Variablen

Die Zielgröße ist die dichotome Variable *Pneumonie* (i15011) wobei *Nein* = 0 und *Ja* = 1 ist.

Die potentiellen erklärenden Variablen sind nachfolgen aufgelistet.

Ordinal skalierte Merkmale

- *Alter*: [0, 45), 45, 55, 65, 75, 85
- *NIHSS* (i4020): [0, 4), 4, 8, 12, 16, 20
- *MRS bei Aufnahme* (i6002): 0, 1, 2, 3, 4, 5
- *MRS vor Insult* (i6001): 0, 1, 2, 3, 4, 5

Aufnahmedaten

- *Geschlecht* (i2005): Männlich, Weiblich
- *Ätiologie* (siehe Definition 1): Mikroangiopathie, Makroangiopathie, Kardiogen Em-bolisch, Andere, Unbekannt
- *Lokalisation* (i7005): Linke Großhirnhemisphäre, Rechte Großhirnhemisphäre, Beide Großhirnhemisphären, Vorwiegend Hirnstamm, Vorwiegend Kleinhirn
- *Klinisches Syndrom* (i7010): LACS, TACS, PACS, POCS, Anderes
- *Zentrum* (nCID): pseudonymisiert

Risikofaktoren

- *Hypertonie*: Nein, Ja, Unbekannt
- *Diabetes mellitus*: Nein, Ja, Unbekannt
- *Vorinsult*: Nein, Ja, Unbekannt
- *Herzinfarkt*: Nein, Ja, Unbekannt
- *Hypercholesterinämie*: Nein, Ja, Unbekannt
- *Vorhofflimmern*: Nein, Ja, Unbekannt
- *Andere kardiale Erkrankung*: Nein, Ja, Unbekannt
- *PAVK*: Nein, Ja, Unbekannt
- *Nikotin*: Nein, Ja, Unbekannt
- *Alkohol regelmäßig*: Nein, Ja, Unbekannt

| 3 MODELL | 13 |
| --- | --- |
|  |  |

Therapie

- *Thrombolyse* (siehe Definition 2): Nein, Ja
- *Heparin (Therapie)* (siehe Definition 3): Keines/Low, Medium, High/iv
- *Thrombozytenaggregationshemmer* (i10005): Nein, Ja
- *Andere zerebrovask. Akutmedikation* (i10006): Nein, Ja
- *Ass. oder kontrollierte Beatmung* (i10007): Nein, Ja
- *Insulin (kontinuierliche i.v. Therapie)* (i10009): Nein, Ja
- *Dopamin (kontinuierliche i.v. Therapie)* (i10010): Nein, Ja
- *Antihypertensiva (kontinuierliche i.v. Therapie)* (i10011): Nein, Ja
- *Intubation* (i10013): Nein, Ja
- *Nasogastrische Sonde* (i10014): Nein, Ja
- *Perkutane Gastrostomie* (i10015): Nein, Ja
- *Blasenkatheter* (i10016): Nein, Ja
- *Op. Dekompression* (i10017): Nein, Ja
- *Physiotherapie* (i10019): Nein, Ja
- *Ergotherapie* (i10020): Nein, Ja
- *Logopädie* (i10021): Nein, Ja

Komplikationen

- *Reinsult* (i15001): Nein, Ja
- *Klin. relevante Einblutung* (i15002): Nein, Ja
- *Hirnödem* (i15003): Nein, Ja
- *Epil. Anfall* (i15004): Nein, Ja
- *Hydrocephalus* (i15005): Nein, Ja
- *Kardiale Arrythmien* (i15006): Nein, Ja
- *Herz-Kreislaufdekompensation* (i15007): Nein, Ja
- *Pulmonalembolie* (i15008): Nein, Ja
- *Sepsis* (i15009): Nein, Ja
- *Harnwegsinfekt* (i15010): Nein, Ja
- *Extrazerebrale Blutung* (i15012): Nein, Ja
- *Tiefe Beinvenenthrombose* (i15013): Nein, Ja
- *Progressive Stroke* (i15014): Nein, Ja
- *Herzinfarkt* (i15016): Nein, Ja

| 3 MODELL | 14 |
| --- | --- |
|  |  |

3.2 Resultierendes Modell

Das Modell wurde durch schrittweise Variablenselektion gefunden wobei das BIC-Kriterium optimiert wurde.

|  | Coef. | SE | z | P value | OR | 95% CI |
| --- | --- | --- | --- | --- | --- | --- |
|  |  |  |  | | |  |
| (Intercept) | -6.16 | 0.15 | -40.11 *<*0.001 0.00 | | | 0 to 0 |
| Blasenkatheter Ja | 0.88 | 0.06 | 14.64 | *<*0.001 | 2.42 | 2.15 to 2.72 |
| Nasogastrische Sonde Ja | 1.14 | 0.05 | 22.65 | *<*0.001 | 3.14 | 2.84 to 3.47 |
| MRS Aufnahme 4 | 0.64 | 0.08 | 8.02 | *<*0.001 | 1.89 | 1.62 to 2.21 |
| Kardiale Arrythmien Ja | 0.73 | 0.08 | 9.34 | *<*0.001 | 2.08 | 1.79 to 2.43 |
| MRS Aufnahme 5 | 0.40 | 0.05 | 7.35 | *<*0.001 | 1.49 | 1.34 to 1.66 |
| Herz-Kreislaufdekomp. Ja | 0.94 | 0.09 | 10.81 | *<*0.001 | 2.56 | 2.16 to 3.04 |
| Geschlecht Weiblich | -0.58 | 0.04 | -12.96 *<*0.001 0.56 | | | 0.51 to 0.61 |
| Alter 75-84 | 0.42 | 0.05 | 8.49 | *<*0.001 | 1.52 | 1.38 to 1.67 |
| Perkutane Gastrostomie Ja | 0.92 | 0.11 | 8.22 | *<*0.001 | 2.52 | 2.02 to 3.14 |
| NIHSS 5-8 | 0.43 | 0.07 | 5.78 | *<*0.001 | 1.53 | 1.33 to 1.77 |
| Zentrum KE | -1.25 | 0.16 | -7.92 | *<*0.001 | 0.29 | 0.21 to 0.39 |
| Zentrum MO | -1.28 | 0.16 | -7.81 *<*0.001 0.28 | | | 0.2 to 0.38 |
| Progressive Stroke Ja | 0.54 | 0.08 | 6.83 | *<*0.001 | 1.72 | 1.47 to 2.01 |
| Zentrum BA | 1.17 | 0.14 | 8.50 | *<*0.001 | 3.22 | 2.46 to 4.22 |
| Sepsis Ja | 1.24 | 0.17 | 7.23 | *<*0.001 | 3.45 | 2.47 to 4.83 |
| Zentrum HI | 0.74 | 0.10 | 7.41 | *<*0.001 | 2.10 | 1.73 to 2.56 |
| Zentrum SA | 1.06 | 0.13 | 8.22 | *<*0.001 | 2.89 | 2.25 to 3.73 |
| Vorhofflimmern Ja | 0.23 | 0.05 | 5.03 | *<*0.001 | 1.26 | 1.15 to 1.38 |
| Logopädie Ja | 0.31 | 0.05 | 6.17 | *<*0.001 | 1.36 | 1.23 to 1.5 |
| Zentrum FU | 0.60 | 0.11 | 5.36 | *<*0.001 | 1.83 | 1.47 to 2.28 |
| NIHSS 13-16 | 0.39 | 0.06 | 6.97 | *<*0.001 | 1.48 | 1.33 to 1.65 |
| Alter 55-64 | 0.65 | 0.11 | 5.84 | *<*0.001 | 1.92 | 1.54 to 2.39 |
| Alkohol regelmäßig Ja | 0.41 | 0.08 | 5.40 | *<*0.001 | 1.51 | 1.3 to 1.75 |
| Zentrum SU | -1.07 | 0.23 | -4.68 | *<*0.001 | 0.34 | 0.22 to 0.54 |
| Zentrum WE | -0.79 | 0.12 | -6.59 *<*0.001 0.45 | | | 0.36 to 0.57 |
| Antihypertensiva (i.v. Therapie) Ja | 0.34 | 0.07 | 4.73 | *<*0.001 | 1.41 | 1.22 to 1.62 |
| Zentrum SI | -2.21 | 0.59 | -3.73 *<*0.001 0.11 | | | 0.03 to 0.35 |
| Hirnödem Ja | 0.45 | 0.10 | 4.52 | *<*0.001 | 1.56 | 1.29 to 1.9 |
| MRS Aufnahme 2 | 0.58 | 0.13 | 4.51 | *<*0.001 | 1.79 | 1.39 to 2.31 |
| Zentrum JA | 0.87 | 0.17 | 5.14 | *<*0.001 | 2.40 | 1.72 to 3.35 |
| Epil. Anfall Ja | 0.55 | 0.13 | 4.39 | *<*0.001 | 1.74 | 1.36 to 2.23 |
| Zentrum LA | -0.34 | 0.09 | -3.78 | *<*0.001 | 0.71 | 0.6 to 0.85 |
| Lokalisation Linke Großhirnhem. | -0.16 | 0.04 | -3.78 *<*0.001 0.85 | | | 0.78 to 0.93 |
| Insulin (i.v. Therapie) Ja | 0.44 | 0.12 | 3.60 | *<*0.001 | 1.55 | 1.22 to 1.98 |
| Harnwegsinfekt Ja | 0.22 | 0.07 | 3.36 | *<*0.001 | 1.25 | 1.1 to 1.42 |
| Ätiologie Unbekannt | -0.17 | 0.05 | -3.35 *<*0.001 0.85 | | | 0.77 to 0.93 |
| Reinsult Ja | 0.52 | 0.15 | 3.45 | *<*0.001 | 1.68 | 1.25 to 2.25 |
|  |  |  |  |  |  |  |

Tabelle 11: N = 59558; Coef.: Koeffizient (coefficient) des Modells, SE: Standardfehler (standard error) der Koeffizienten, z: Prüfstatistik, P value: P-Wert des Signifi-kanztests H0 Coef = 0, OR: Odds Ratio, 95% CI: 95% Konfidenzintervall (con-fidence interval) der OR

| 3 MODELL | | 15 | | |  |
| --- | --- | --- | --- | --- | --- |
|  |  |  |  |  |  |
|  |  |  |  |  |  |
|  | N | 59558 |  |  |  |
|  | Anzahl Koeffizienten | 38 | |  |  |
|  | McKelvey-Zavoina Pseudo-*R*^2^ | 0.46 | |  |  |
|  | Nagelkerke Pseudo-*R*^2^ | 0.35 | |  |  |
|  | McFadden Pseudo-*R*^2^ | 0.3 | |  |  |
|  | Maximum likelihood Pseudo-*R*^2^ | 0.12 | |  |  |
|  | Cragg and Uhler’s Pseudo-*R*^2^ | 0.35 | |  |  |

Tabelle 12: Kennzahlen des Logit-Modells

| 3 MODELL | |  |  |  |  |  | 16 | |  |
| --- | --- | --- | --- | --- | --- | --- | --- | --- | --- |
|  |  |  |  |  |  |  |  |  |  |
|  |  |  |  |  |  |  |  |  |  |
|  |  | Coef. | SE | z | P value | OR | 95% CI |  |  |
|  | (Intercept) | -6.16 | 0.15 | -40.11 | *<*0.001 | 0.00 | 0 to 0 |  |  |
|  | Geschlecht Weiblich | -0.58 | 0.04 | -12.96 *<*0.001 0.56 | | | 0.51 to 0.61 | |  |
|  | Alter 55-64 | 0.65 | 0.11 | 5.84 | *<*0.001 | 1.92 | 1.54 to 2.39 | |  |
|  | Alter 75-84 | 0.42 | 0.05 | 8.49 | *<*0.001 | 1.52 | 1.38 to 1.67 | |  |
|  | NIHSS 5-8 | 0.43 | 0.07 | 5.78 | *<*0.001 | 1.53 | 1.33 to 1.77 |  |  |
|  | NIHSS 13-16 | 0.39 | 0.06 | 6.97 | *<*0.001 | 1.48 | 1.33 to 1.65 | |  |
|  | MRS Aufnahme 2 | 0.58 | 0.13 | 4.51 | *<*0.001 | 1.79 | 1.39 to 2.31 | |  |
|  | MRS Aufnahme 4 | 0.64 | 0.08 | 8.02 | *<*0.001 | 1.89 | 1.62 to 2.21 | |  |
|  | MRS Aufnahme 5 | 0.40 | 0.05 | 7.35 | *<*0.001 | 1.49 | 1.34 to 1.66 | |  |
|  | Ätiologie Unbekannt | -0.17 | 0.05 | -3.35 | *<*0.001 | 0.85 | 0.77 to 0.93 |  |  |
|  | Lokalisation Linke Großhirnhem. | -0.16 | 0.04 | -3.78 *<*0.001 0.85 | | | 0.78 to 0.93 | |  |
|  |  |  |  |  |  |  |  |  |  |
|  | Vorhofflimmern Ja | 0.23 | 0.05 | 5.03 | *<*0.001 | 1.26 | 1.15 to 1.38 |  |  |
|  | Alkohol regelmäßig Ja | 0.41 | 0.08 | 5.40 | *<*0.001 | 1.51 | 1.3 to 1.75 | |  |
|  |  |  |  |  |  |  |  |  |  |
|  | Insulin (i.v. Therapie) Ja | 0.44 | 0.12 | 3.60 | *<*0.001 | 1.55 | 1.22 to 1.98 |  |  |
|  | Antihypertensiva (i.v. Therapie) Ja | 0.34 | 0.07 | 4.73 | *<*0.001 | 1.41 | 1.22 to 1.62 | |  |
|  | Nasogastrische Sonde Ja | 1.14 | 0.05 | 22.65 | *<*0.001 | 3.14 | 2.84 to 3.47 | |  |
|  | Perkutane Gastrostomie Ja | 0.92 | 0.11 | 8.22 | *<*0.001 | 2.52 | 2.02 to 3.14 | |  |
|  | Blasenkatheter Ja | 0.88 | 0.06 | 14.64 | *<*0.001 | 2.42 | 2.15 to 2.72 | |  |
|  | Logopädie Ja | 0.31 | 0.05 | 6.17 | *<*0.001 | 1.36 | 1.23 to 1.5 | |  |
|  |  |  |  |  |  |  |  |  |  |
|  | Reinsult Ja | 0.52 | 0.15 | 3.45 | *<*0.001 | 1.68 | 1.25 to 2.25 |  |  |
|  | Hirnödem Ja | 0.45 | 0.10 | 4.52 | *<*0.001 | 1.56 | 1.29 to 1.9 | |  |
|  | Epil. Anfall Ja | 0.55 | 0.13 | 4.39 | *<*0.001 | 1.74 | 1.36 to 2.23 | |  |
|  | Kardiale Arrythmien Ja | 0.73 | 0.08 | 9.34 | *<*0.001 | 2.08 | 1.79 to 2.43 | |  |
|  | Herz-Kreislaufdekomp. Ja | 0.94 | 0.09 | 10.81 | *<*0.001 | 2.56 | 2.16 to 3.04 | |  |
|  | Sepsis Ja | 1.24 | 0.17 | 7.23 | *<*0.001 | 3.45 | 2.47 to 4.83 | |  |
|  | Harnwegsinfekt Ja | 0.22 | 0.07 | 3.36 | *<*0.001 | 1.25 | 1.1 to 1.42 | |  |
|  | Progressive Stroke Ja | 0.54 | 0.08 | 6.83 | *<*0.001 | 1.72 | 1.47 to 2.01 | |  |
|  |  |  |  |  |  |  |  |  |  |
|  | Zentrum KE | -1.25 | 0.16 | -7.92 | *<*0.001 | 0.29 | 0.21 to 0.39 |  |  |
|  | Zentrum MO | -1.28 | 0.16 | -7.81 *<*0.001 0.28 | | | 0.2 to 0.38 | |  |
|  | Zentrum BA | 1.17 | 0.14 | 8.50 | *<*0.001 | 3.22 | 2.46 to 4.22 | |  |
|  | Zentrum HI | 0.74 | 0.10 | 7.41 | *<*0.001 | 2.10 | 1.73 to 2.56 | |  |
|  | Zentrum SA | 1.06 | 0.13 | 8.22 | *<*0.001 | 2.89 | 2.25 to 3.73 | |  |
|  | Zentrum FU | 0.60 | 0.11 | 5.36 | *<*0.001 | 1.83 | 1.47 to 2.28 | |  |
|  | Zentrum SU | -1.07 | 0.23 | -4.68 | *<*0.001 | 0.34 | 0.22 to 0.54 | |  |
|  | Zentrum WE | -0.79 | 0.12 | -6.59 | *<*0.001 | 0.45 | 0.36 to 0.57 | |  |
|  | Zentrum SI | -2.21 | 0.59 | -3.73 *<*0.001 0.11 | | | 0.03 to 0.35 | |  |
|  | Zentrum JA | 0.87 | 0.17 | 5.14 | *<*0.001 | 2.40 | 1.72 to 3.35 | |  |
|  | Zentrum LA | -0.34 | 0.09 | -3.78 | *<*0.001 | 0.71 | 0.6 to 0.85 | |  |
|  |  |  |  |  |  |  |  |  |  |

Tabelle 13: Koeffizienten des Modells, inhaltlich geordnet

| 3 | MODELL |  |  |  | 17 |
| --- | --- | --- | --- | --- | --- |
|  | Female sex |  |  |  |  |
|  | Age 55−64 |  |  |  |  |
|  | Age 75−84 |  |  |  |  |
|  | NIHSS >= 5 |  |  |  |  |
|  | NIHSS >= 13 |  |  |  |  |
|  | MRS >= 2 |  |  |  |  |
|  | MRS >= 4 |  |  |  |  |
|  | MRS >= 5 |  |  |  |  |
|  | Cryptogenic stroke |  |  |  |  |
|  | Left hemispheric stroke |  |  |  |  |
|  | Atrial fibrillation |  |  |  |  |
|  | Regular alcohol consumption |  |  |  |  |
|  | Continuous iv insulin |  |  |  |  |
|  | Iv antihypertensive therapy |  |  |  |  |
|  | Nasogastric tube |  |  |  |  |
| Percutaneous endoscopic gastrostomy | |  |  |  |  |
|  | Urinary catheter |  |  |  |  |
|  | Speech and language therapy |  |  |  |  |
|  | Recurrent stroke |  |  |  |  |
|  | Cerebral edema |  |  |  |  |
|  | Epileptic seizure |  |  |  |  |
|  | Arrhythmia |  |  |  |  |
|  | Heart failure |  |  |  |  |
|  | Septicemia |  |  |  |  |
|  | Urinary tract infection |  |  |  |  |
|  | Progressive Stroke |  |  |  |  |
|  | 0 | 1 | 2 | 3 | 4 |
|  |  |  | Odds−Ratio |  |  |


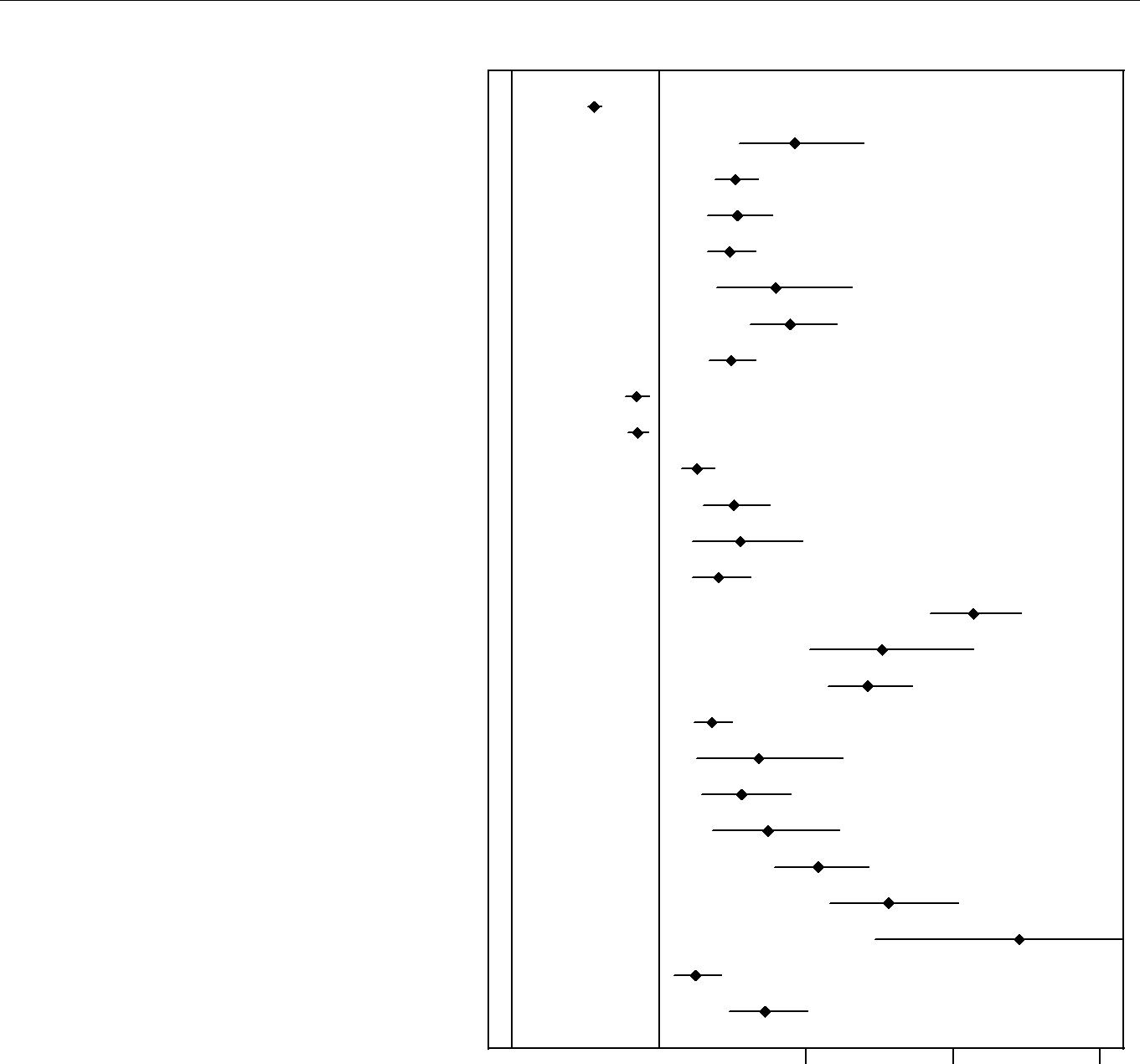


Abbildung 1: Odds-Ratios mit 95 % CI

| 3 | MODELL |  |  |  |  | 18 |
| --- | --- | --- | --- | --- | --- | --- |
|  | Female sex |  |  |  |  |  |
|  | Age 55−64 |  |  |  |  |  |
|  | Age 75−84 |  |  |  |  |  |
|  | NIHSS >= 5 |  |  |  |  |  |
|  | NIHSS >= 13 |  |  |  |  |  |
|  | MRS >= 2 |  |  |  |  |  |
|  | MRS >= 4 |  |  |  |  |  |
|  | MRS >= 5 |  |  |  |  |  |
|  | Cryptogenic stroke |  |  |  |  |  |
|  | Left hemispheric stroke |  |  |  |  |  |
|  | Atrial fibrillation |  |  |  |  |  |
|  | Regular alcohol consumption |  |  |  |  |  |
|  | Continuous iv insulin |  |  |  |  |  |
|  | Iv antihypertensive therapy |  |  |  |  |  |
|  | Nasogastric tube |  |  |  |  |  |
| Percutaneous endoscopic gastrostomy | |  |  |  |  |  |
|  | Urinary catheter |  |  |  |  |  |
|  | Speech and language therapy |  |  |  |  |  |
|  | Recurrent stroke |  |  |  |  |  |
|  | Cerebral edema |  |  |  |  |  |
|  | Epileptic seizure |  |  |  |  |  |
|  | Arrhythmia |  |  |  |  |  |
|  | Heart failure |  |  |  |  |  |
|  | Septicemia |  |  |  |  |  |
|  | Urinary tract infection |  |  |  |  |  |
|  | Progressive Stroke |  |  |  |  |  |
|  | 0 | 1 | 2 | 3 | 4 | 5 |
|  |  |  |  | Odds−Ratio |  |  |


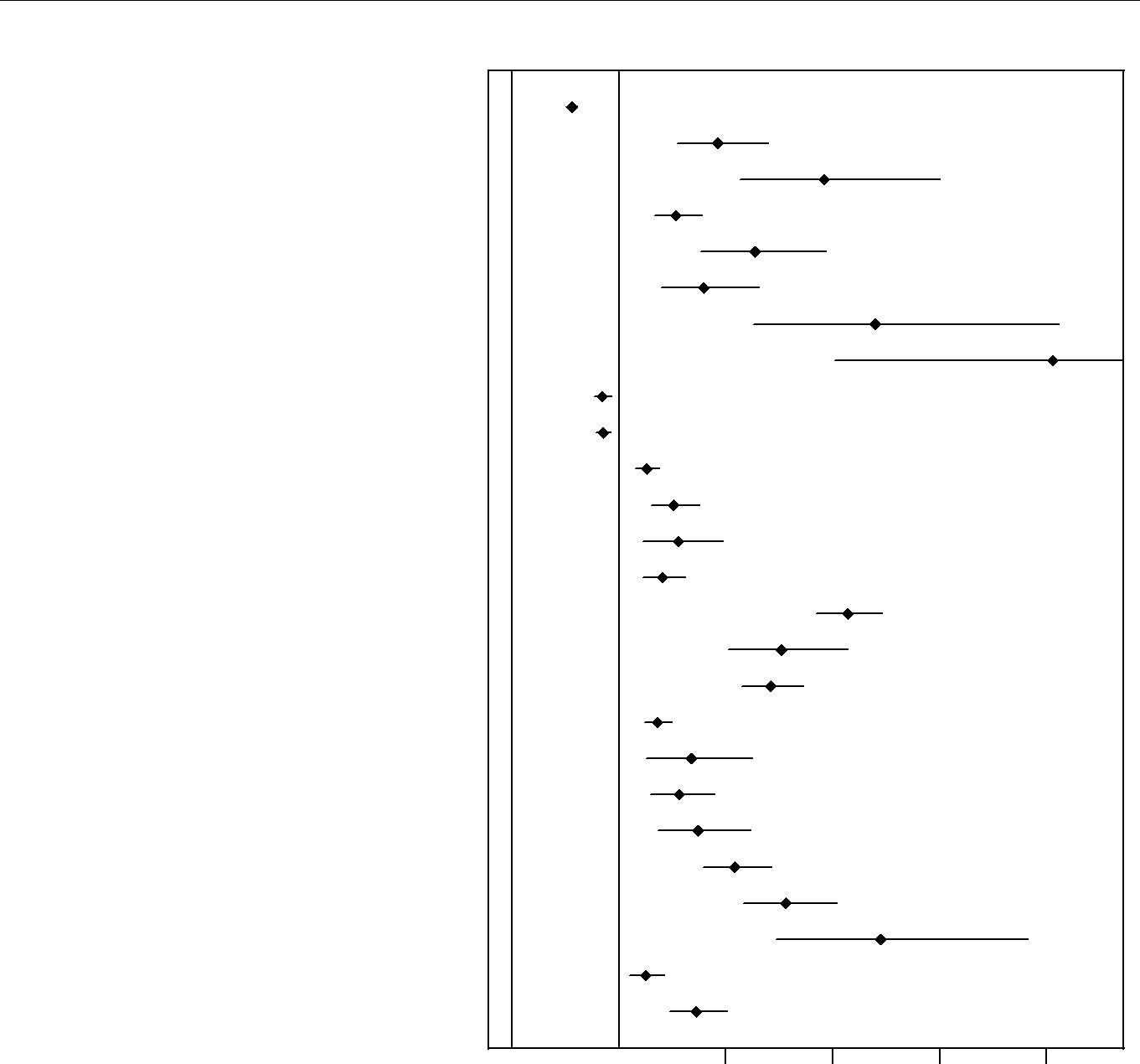


Abbildung 2: Odds-Ratios mit 95 % CI; bei Alter, NIHSS und MRS sind für die höheren Ausprägungen bereits die aggregierten OR angegeben.

| 3 MODELL | 19 |
| --- | --- |
|  |  |

3.3 Resultierendes Modell 2

Analog zum vorigen Abschnitt wobei aber nur die 47321 Fälle mit vorliegender Eingabe von *Lipidsenker* (i17021) genommen wurden.

|  | Coef. | SE | z | P value | OR | 95% CI |
| --- | --- | --- | --- | --- | --- | --- |
|  |  |  |  | | |  |
| (Intercept) | -6.18 | 0.17 | -36.24 *<*0.001 0.00 | | | 0 to 0 |
| Blasenkatheter Ja | 0.84 | 0.07 | 12.54 | *<*0.001 | 2.31 | 2.03 to 2.64 |
| Nasogastrische Sonde Ja | 1.18 | 0.06 | 20.64 | *<*0.001 | 3.26 | 2.91 to 3.64 |
| MRS Aufnahme 4 | 0.62 | 0.09 | 6.93 | *<*0.001 | 1.86 | 1.56 to 2.21 |
| Kardiale Arrythmien Ja | 0.83 | 0.09 | 8.86 | *<*0.001 | 2.30 | 1.92 to 2.77 |
| MRS Aufnahme 5 | 0.42 | 0.06 | 6.96 | *<*0.001 | 1.52 | 1.35 to 1.72 |
| Herz-Kreislaufdekomp. Ja | 0.91 | 0.11 | 8.55 | *<*0.001 | 2.47 | 2.01 to 3.05 |
| Geschlecht Weiblich | -0.56 | 0.05 | -11.03 *<*0.001 0.57 | | | 0.52 to 0.63 |
| Alter 75-84 | 0.35 | 0.06 | 6.32 | *<*0.001 | 1.42 | 1.28 to 1.59 |
| Perkutane Gastrostomie Ja | 0.86 | 0.13 | 6.40 | *<*0.001 | 2.35 | 1.81 to 3.06 |
| NIHSS 5-8 | 0.53 | 0.08 | 6.33 | *<*0.001 | 1.70 | 1.44 to 2 |
| Zentrum KE | -1.32 | 0.21 | -6.13 | *<*0.001 | 0.27 | 0.18 to 0.41 |
| Zentrum MO | -1.44 | 0.20 | -7.10 *<*0.001 0.24 | | | 0.16 to 0.35 |
| Progressive Stroke Ja | 0.62 | 0.09 | 7.00 | *<*0.001 | 1.86 | 1.57 to 2.22 |
| Zentrum BA | 1.21 | 0.14 | 8.55 | *<*0.001 | 3.34 | 2.53 to 4.41 |
| Sepsis Ja | 1.02 | 0.20 | 5.02 | *<*0.001 | 2.79 | 1.87 to 4.15 |
| Zentrum HI | 0.89 | 0.11 | 8.16 | *<*0.001 | 2.45 | 1.97 to 3.03 |
| Zentrum SA | 1.21 | 0.13 | 9.11 | *<*0.001 | 3.34 | 2.58 to 4.33 |
| Vorhofflimmern Ja | 0.28 | 0.05 | 5.60 | *<*0.001 | 1.33 | 1.2 to 1.47 |
| Logopädie Ja | 0.37 | 0.06 | 6.00 | *<*0.001 | 1.44 | 1.28 to 1.62 |
| Zentrum FU | 0.85 | 0.12 | 6.90 | *<*0.001 | 2.33 | 1.83 to 2.97 |
| NIHSS 13-16 | 0.37 | 0.06 | 5.93 | *<*0.001 | 1.45 | 1.28 to 1.64 |
| Alter 55-64 | 0.68 | 0.13 | 5.37 | *<*0.001 | 1.98 | 1.54 to 2.54 |
| Alkohol regelmäßig Ja | 0.44 | 0.09 | 5.08 | *<*0.001 | 1.55 | 1.31 to 1.84 |
| Zentrum SU | -1.00 | 0.23 | -4.40 *<*0.001 0.37 | | | 0.23 to 0.57 |
| Antihypertensiva (i.v. Therapie) Ja | 0.46 | 0.08 | 5.73 | *<*0.001 | 1.58 | 1.35 to 1.84 |
| Zentrum SI | -2.15 | 0.59 | -3.62 *<*0.001 0.12 | | | 0.04 to 0.37 |
| MRS Aufnahme 2 | 0.49 | 0.14 | 3.42 | *<*0.001 | 1.63 | 1.23 to 2.15 |
| Zentrum JA | 0.88 | 0.17 | 5.16 | *<*0.001 | 2.42 | 1.73 to 3.39 |
| Epil. Anfall Ja | 0.57 | 0.15 | 3.88 | *<*0.001 | 1.77 | 1.32 to 2.35 |
| Lokalisation Linke Großhirnhem. | -0.16 | 0.05 | -3.28 | 0.001 | 0.86 | 0.78 to 0.94 |
| Lipidsenker Ja | -0.23 | 0.05 | -4.79 | *<*0.001 | 0.79 | 0.72 to 0.87 |
| Zentrum RA | -0.59 | 0.13 | -4.51 *<*0.001 0.55 | | | 0.43 to 0.72 |
| And. kard. Erkrankung Ja | 0.20 | 0.05 | 3.86 | *<*0.001 | 1.22 | 1.1 to 1.35 |
| Zentrum WA | 0.49 | 0.14 | 3.62 | *<*0.001 | 1.63 | 1.25 to 2.13 |
|  |  |  |  |  |  |  |

Tabelle 14: N = 47321; Coef.: Koeffizient (coefficient) des Modells, SE: Standardfehler (standard error) der Koeffizienten, z: Prüfstatistik, P value: P-Wert des Signifi-kanztests H0 Coef = 0, OR: Odds Ratio, 95% CI: 95% Konfidenzintervall (con-fidence interval) der OR

| 3 MODELL | | 20 | | |  |
| --- | --- | --- | --- | --- | --- |
|  |  |  |  |  |  |
|  |  |  |  |  |  |
|  | N | 47321 |  |  |  |
|  | Anzahl Koeffizienten | 35 | |  |  |
|  | McKelvey-Zavoina Pseudo-*R*^2^ | 0.46 | |  |  |
|  | Nagelkerke Pseudo-*R*^2^ | 0.34 | |  |  |
|  | McFadden Pseudo-*R*^2^ | 0.3 | |  |  |
|  | Maximum likelihood Pseudo-*R*^2^ | 0.11 | |  |  |
|  | Cragg and Uhler’s Pseudo-*R*^2^ | 0.34 | |  |  |

Tabelle 15: Kennzahlen des Logit-Modells

|  | Coef. | SE | z | P value | OR | 95% CI |
| --- | --- | --- | --- | --- | --- | --- |
|  |  |  |  |  |  |  |
| (Intercept) | -6.18 | 0.17 | -36.24 | *<*0.001 | 0.00 | 0 to 0 |
| Geschlecht Weiblich | -0.56 | 0.05 | -11.03 *<*0.001 0.57 | | | 0.52 to 0.63 |
| Alter 55-64 | 0.68 | 0.13 | 5.37 | *<*0.001 | 1.98 | 1.54 to 2.54 |
| Alter 75-84 | 0.35 | 0.06 | 6.32 | *<*0.001 | 1.42 | 1.28 to 1.59 |
| NIHSS 5-8 | 0.53 | 0.08 | 6.33 | *<*0.001 | 1.70 | 1.44 to 2 |
| NIHSS 13-16 | 0.37 | 0.06 | 5.93 | *<*0.001 | 1.45 | 1.28 to 1.64 |
| MRS Aufnahme 2 | 0.49 | 0.14 | 3.42 | *<*0.001 | 1.63 | 1.23 to 2.15 |
| MRS Aufnahme 4 | 0.62 | 0.09 | 6.93 | *<*0.001 | 1.86 | 1.56 to 2.21 |
| MRS Aufnahme 5 | 0.42 | 0.06 | 6.96 | *<*0.001 | 1.52 | 1.35 to 1.72 |
| Lokalisation Linke Großhirnhem. | -0.16 | 0.05 | -3.28 | 0.001 | 0.86 | 0.78 to 0.94 |
| And. kard. Erkrankung Ja | 0.20 | 0.05 | 3.86 | *<*0.001 | 1.22 | 1.1 to 1.35 |
| Vorhofflimmern Ja | 0.28 | 0.05 | 5.60 | *<*0.001 | 1.33 | 1.2 to 1.47 |
| Alkohol regelmäßig Ja | 0.44 | 0.09 | 5.08 | *<*0.001 | 1.55 | 1.31 to 1.84 |
|  |  |  |  |  |  |  |
| Antihypertensiva (i.v. Therapie) Ja | 0.46 | 0.08 | 5.73 | *<*0.001 | 1.58 | 1.35 to 1.84 |
| Nasogastrische Sonde Ja | 1.18 | 0.06 | 20.64 | *<*0.001 | 3.26 | 2.91 to 3.64 |
| Perkutane Gastrostomie Ja | 0.86 | 0.13 | 6.40 | *<*0.001 | 2.35 | 1.81 to 3.06 |
| Blasenkatheter Ja | 0.84 | 0.07 | 12.54 | *<*0.001 | 2.31 | 2.03 to 2.64 |
| Logopädie Ja | 0.37 | 0.06 | 6.00 | *<*0.001 | 1.44 | 1.28 to 1.62 |
|  |  |  |  |  |  |  |
| Epil. Anfall Ja | 0.57 | 0.15 | 3.88 | *<*0.001 | 1.77 | 1.32 to 2.35 |
| Kardiale Arrythmien Ja | 0.83 | 0.09 | 8.86 | *<*0.001 | 2.30 | 1.92 to 2.77 |
| Herz-Kreislaufdekomp. Ja | 0.91 | 0.11 | 8.55 | *<*0.001 | 2.47 | 2.01 to 3.05 |
| Sepsis Ja | 1.02 | 0.20 | 5.02 | *<*0.001 | 2.79 | 1.87 to 4.15 |
| Progressive Stroke Ja | 0.62 | 0.09 | 7.00 | *<*0.001 | 1.86 | 1.57 to 2.22 |
|  |  |  |  |  |  |  |
| Lipidsenker Ja | -0.23 | 0.05 | -4.79 | *<*0.001 | 0.79 | 0.72 to 0.87 |
| Zentrum KE | -1.32 | 0.21 | -6.13 | *<*0.001 | 0.27 | 0.18 to 0.41 |
| Zentrum MO | -1.44 | 0.20 | -7.10 *<*0.001 0.24 | | | 0.16 to 0.35 |
| Zentrum BA | 1.21 | 0.14 | 8.55 | *<*0.001 | 3.34 | 2.53 to 4.41 |
| Zentrum HI | 0.89 | 0.11 | 8.16 | *<*0.001 | 2.45 | 1.97 to 3.03 |
| Zentrum SA | 1.21 | 0.13 | 9.11 | *<*0.001 | 3.34 | 2.58 to 4.33 |
| Zentrum FU | 0.85 | 0.12 | 6.90 | *<*0.001 | 2.33 | 1.83 to 2.97 |
| Zentrum SU | -1.00 | 0.23 | -4.40 | *<*0.001 | 0.37 | 0.23 to 0.57 |
| Zentrum SI | -2.15 | 0.59 | -3.62 *<*0.001 0.12 | | | 0.04 to 0.37 |
| Zentrum JA | 0.88 | 0.17 | 5.16 | *<*0.001 | 2.42 | 1.73 to 3.39 |
| Zentrum RA | -0.59 | 0.13 | -4.51 *<*0.001 0.55 | | | 0.43 to 0.72 |
| Zentrum WA | 0.49 | 0.14 | 3.62 | *<*0.001 | 1.63 | 1.25 to 2.13 |
|  |  |  |  |  |  |  |

Tabelle 16: Koeffizienten des Modells, inhaltlich geordnet

| 3 | MODELL |  |  |  | 21 |
| --- | --- | --- | --- | --- | --- |
|  | Female sex |  |  |  |  |
|  | Age 55−64 |  |  |  |  |
|  | Age 75−84 |  |  |  |  |
|  | NIHSS >= 5 |  |  |  |  |
|  | NIHSS >= 13 |  |  |  |  |
|  | MRS >= 2 |  |  |  |  |
|  | MRS >= 4 |  |  |  |  |
|  | MRS >= 5 |  |  |  |  |
|  | Left hemispheric stroke |  |  |  |  |
|  | Non−ischemic heart disease |  |  |  |  |
|  | Atrial fibrillation |  |  |  |  |
|  | Regular alcohol consumption |  |  |  |  |
|  | Iv antihypertensive therapy |  |  |  |  |
|  | Nasogastric tube |  |  |  |  |
| Percutaneous endoscopic gastrostomy | |  |  |  |  |
|  | Urinary catheter |  |  |  |  |
|  | Speech and language therapy |  |  |  |  |
|  | Epileptic seizure |  |  |  |  |
|  | Arrhythmia |  |  |  |  |
|  | Heart failure |  |  |  |  |
|  | Septicemia |  |  |  |  |
|  | Progressive Stroke |  |  |  |  |
|  | Lipid lowering therapy |  |  |  |  |
|  | 0 | 1 | 2 | 3 | 4 |
|  |  |  | Odds−Ratio |  |  |


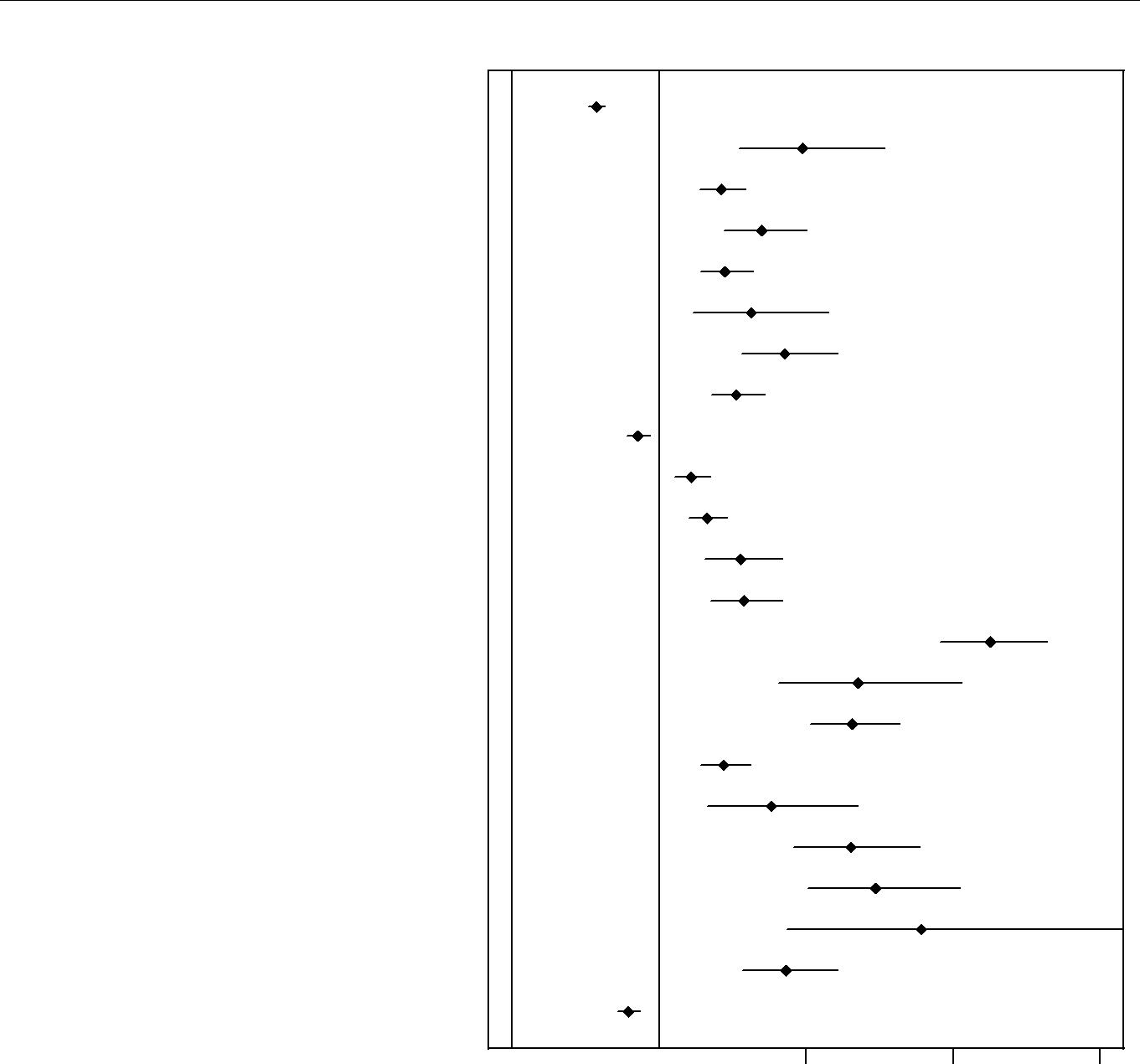


Abbildung 3: Odds-Ratios mit 95 % CI

| 3 | MODELL |  |  |  |  | 22 |
| --- | --- | --- | --- | --- | --- | --- |
|  | Female sex |  |  |  |  |  |
|  | Age 55−64 |  |  |  |  |  |
|  | Age 75−84 |  |  |  |  |  |
|  | NIHSS >= 5 |  |  |  |  |  |
|  | NIHSS >= 13 |  |  |  |  |  |
|  | MRS >= 2 |  |  |  |  |  |
|  | MRS >= 4 |  |  |  |  |  |
|  | MRS >= 5 |  |  |  |  |  |
|  | Left hemispheric stroke |  |  |  |  |  |
|  | Non−ischemic heart disease |  |  |  |  |  |
|  | Atrial fibrillation |  |  |  |  |  |
|  | Regular alcohol consumption |  |  |  |  |  |
|  | Iv antihypertensive therapy |  |  |  |  |  |
|  | Nasogastric tube |  |  |  |  |  |
| Percutaneous endoscopic gastrostomy | |  |  |  |  |  |
|  | Urinary catheter |  |  |  |  |  |
|  | Speech and language therapy |  |  |  |  |  |
|  | Epileptic seizure |  |  |  |  |  |
|  | Arrhythmia |  |  |  |  |  |
|  | Heart failure |  |  |  |  |  |
|  | Septicemia |  |  |  |  |  |
|  | Progressive Stroke |  |  |  |  |  |
|  | Lipid lowering therapy |  |  |  |  |  |
|  | 0 | 1 | 2 | 3 | 4 | 5 |
|  |  |  | Odds−Ratio | |  |  |


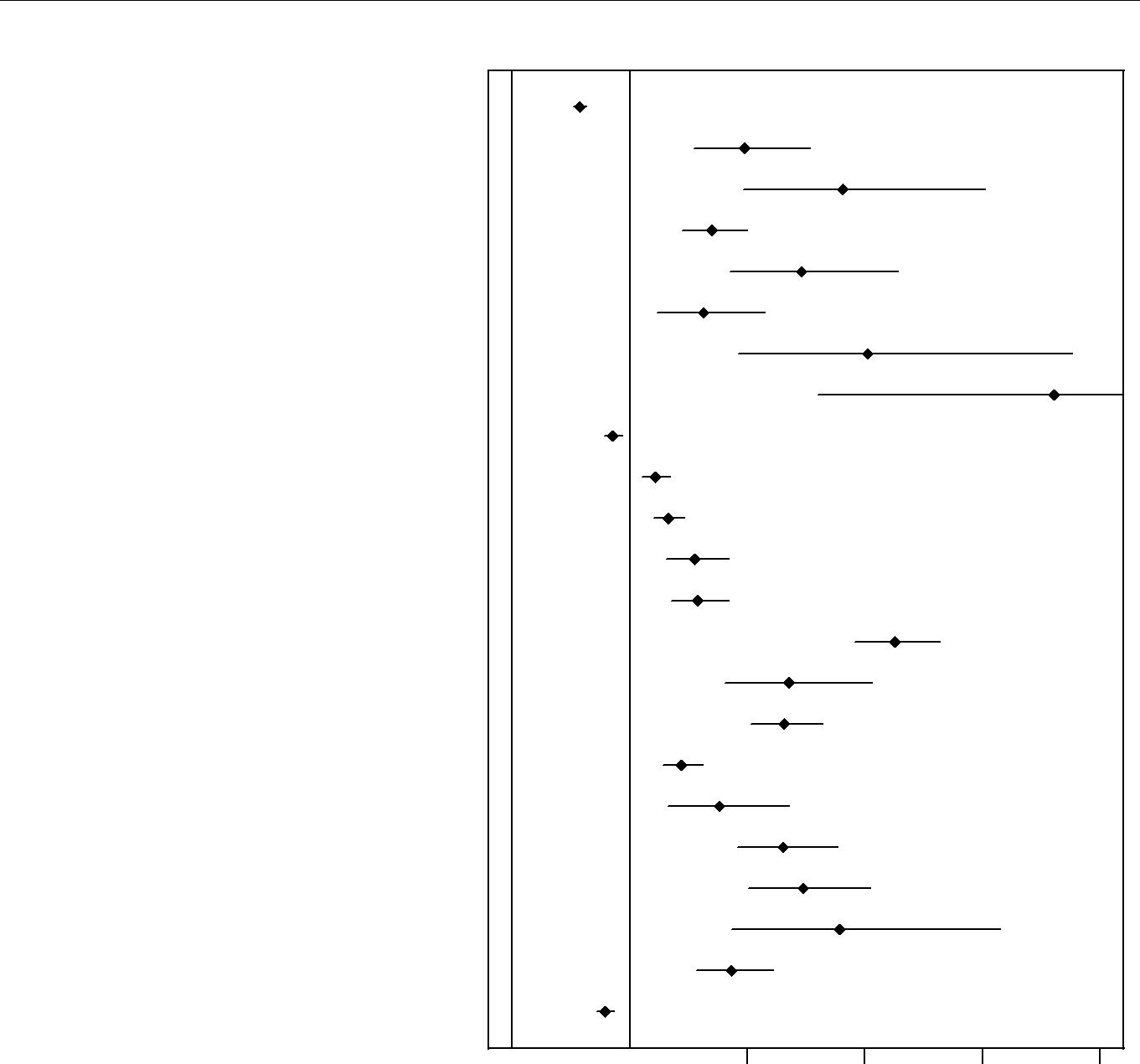


Abbildung 4: Odds-Ratios mit 95 % CI; bei Alter, NIHSS und MRS sind für die höheren Ausprägungen bereits die aggregierten OR angegeben.
