# Supplementary material for: Post-stroke pneumonia at the stroke unit – a registry based analysis of contributing and protective factors
Source: BMC Neurol. 2016 Jul 18;16:107. doi: 10.1186/s12883-016-0627-y (PMC4949772; doi:10.1186/s12883-016-0627-y)
Supplement: Additional file 3: — “Three months mortality”, contains results of multivariate model on study patients with complete data on three months follow up, with mortality as dependent variable. (DOCX 82 kb) [file 12883_2016_627_MOESM3_ESM.docx]

**Mortality at three months** – multivariate model with mortality as dependent variable, patients with complete three months follow up, n=24136; Coef: model coefficient; SE standard error, z test statistic, OR odds ratio, 95%CI confidence interval of OR
